# PET-Driven Fluorescence Modulation in Halochromic Styryl Hemicyanine Dyes Targeting DNA Minor Groove

**DOI:** 10.3390/molecules30234607

**Published:** 2025-11-30

**Authors:** Teodora Aleksandrova, Aleksandar Pashev, Sonia Ilieva, Raimundo Gargallo, Diana Cheshmedzhieva, Aleksey Vasilev

**Affiliations:** 1 Department of Chemistry and Biochemistry, Faculty of Pharmacy, Medical University Pleven, 1 St. Kliment Ohridski Street, 5800 Pleven, Bulgaria; teodora.aleksandrova@mu-pleven.bg; 2 Faculty of Chemistry and Pharmacy, Sofia University “St. Kliment Ohridski”, 1 James Bourchier Ave., 1164 Sofia, Bulgaria; silieva@chem.uni-sofia.bg; 3 Department of Chemical Engineering and Analytical Chemistry, University of Barcelona, Martí i Franqués 1-11, E-08028 Barcelona, Spain; raimon_gargallo@ub.edu; 4 Institute of Polymers, Bulgarian Academy of Sciences, Akad. G. Bonchev St. bl 103A, 1113 Sofia, Bulgaria

**Keywords:** styryl hemicyanine, dsDNA, PET effect, DFT, molecular docking, solvatochromism

## Abstract

A new series of styryl hemicyanine dyes featuring substituted *N*-phenylpiperazine end groups was synthesized using an environmentally friendly procedure. The photophysical properties of the dyes were systematically investigated in organic solvents of varying polarity and when bound to DNA, using a combination of spectroscopic techniques. The dyes show strong negative solvatochromism and exhibit fluorescence quenching upon DNA binding. The dyes are definitely halochromic, exhibiting pronounced fluorescent acidochromism, accompanied by a photoinduced electron transfer (PET) effect. Titration with acid of the dye–DNA complexes restores fluorescence, indicating suppression of the PET and, at the same time, rigidizing of the chemical structure. UV/VIS and fluorescence titration, circular dichroism spectroscopy, and molecular docking methods were used to investigate the interaction mode between the dyes and DNA. Density functional theory (DFT) and time-dependent density functional theory (TDDFT) quantum chemical calculations were employed in deciphering the observed spectroscopic behavior and PET-related effects. The obtained results suggest the dyes’ potential as pH-responsive fluorescent probes for nucleic acid environments.

## 1. Introduction

In recent years, there has been an increasing interest in *N*-alkylpiperazines and substituted *N*-phenylpiperazines as pharmacophoric groups for a wide range of medical applications. Sirim et al. reported a series of benzimidazole–acrylonitrile derivatives designed and synthesized as new anti-tuberculosis agents [1]. Among them, the one with the *N*-*p*-tolylpiperazine fragment is the most active compound with a MIC of 0.78 mg/mL against *M. tuberculosis*, and therefore more effective than the standard drug ethambutol in terms of MIC. It has been found that the new compound is more effective than the first-line drugs such as isoniazid, ciprofloxacin, rifampicin, and moxifloxacin against nutrient-starved *M. tuberculosis*. This finding indicates that the compound represents a promising lead candidate for the development of therapeutics effective against both active and dormant forms of *M. tuberculosis* [1]. In another study, Mishra and coworkers have successfully designed and synthesized a novel series of 2-(4-(4-substituted piperazin-1-yl) benzylidene)-1*H*-indene-1,3(2*H*)-dione derivatives as potent multitarget agents for the treatment of Alzheimer’s disease [2]. The synthesized derivatives exhibited pronounced inhibitory activity against acetylcholinesterase (AChE) and amyloid-β (Aβ) aggregation. The compounds containing 1-(furan-2-yl)ethan-1-one-piperazine and 2-methylpiperimidinyl-piperazine pharmacophores were found to be the most active inhibitors in the entire series. They selectively inhibited AChE over BuChE and also demonstrated superior inhibition of Aβ aggregation compared to the standard drug Donepezil [2]. In the context of the theranostic potential of organic compounds, Kurutos et al. recently reported the successful development and characterization of a series of piperazine-tethered, pH-responsive cyanine dyes for biological applications [3].

Styryl cyanine dyes are a subclass of cyanine dyes first discovered by Barbier and co-workers in 1920 [4], Koenig and Treichel in 1921 [5], and Mills and co-workers in 1922 [6]. Their original most significant application was as sensitizers for photographic plates [6]. The successful application in this field led to their rapid development as a subclass over the past sixty years. Systematic studies by Ephardt and Fromherz [7], Yarmolyuk [8,9,10,11], Shindy [12], and Wang [13] demonstrated the ability of styryl dyes to be used also as fluorogenic markers for binding biopolymers such as DNA, RNA, and proteins. Recently, Wang et al. successfully demonstrated the capacity of styryl dyes as theragnostic agents [13]. Our interest in the area of biolabeling directed us to find unsymmetrical styryl dyes consisting of benzothiazole and *N*-methylpiperazine or *N*-phenylpiperazine scaffolds with excellent binding affinities for different ds-polynucleotides and G-quadruplexes [14]. It was demonstrated that the substitution of the piperazine unit with a methyl or phenyl group significantly influenced the dyes’ binding modes, binding affinities, spectroscopic responses, and antiproliferative activities [14]. In a previous study, the dual mechanism of fluorescence quenching of an *N*-methylpiperazine-substituted styryl cyanine analog was demonstrated [15]. The main fluorescence quenching factor is the rotational and vibrational motions of the dye molecule in solution [15]. Under acidic conditions, protonation of the nitrogen atoms suppresses the photoinduced electron transfer (PET) process, leading to fluorescence recovery. Therefore, a distinct fluorescent response can be observed only when the dye interacts with DNA and a proton simultaneously. These interesting observations [14,15] stimulated us in the design of new analogs and the investigation of their photophysical properties in the presence of DNA under acidic conditions. Dyes that label tumor cells and exhibit acidochromism are particularly useful in cancer diagnostics and imaging, because they can respond to the acidic microenvironment of tumors. In this work, we further explore how specific substituents in *N*-phenylpiperazine group would affect the photophysical properties and biding affinity of the dyes.

Compounds with *N*-methylpiperazine substituents showed a significant preference for AT-DNA polynucleotides and demonstrated AT-minor groove binding at pH = 5.0, which manifested in strong fluorescence increase, significant double helix stabilization, and positive induced circular dichroism spectra [14]. These compounds formed complexes with G-quadruplex by *π*-*π* stacking interactions of the dye with the top or bottom G-tetrad [14]. Bulkier compounds with *N*-phenylpiperazine function are probably bound to ds-polynucleotide by partial intercalation between base pairs. On the other hand, they showed stronger stabilization of G-quadruplex compared to methyl-substituted compounds [14]. As previously noted, these dyes exhibit interesting pH-dependent photophysical behavior [15], which warrants more detailed investigation. To better understand the influence of the *N*-phenylpiperazine moiety on the photophysical and biological properties of styryl hemicyanine dyes, we have synthesized a series of novel derivatives featuring various substituents in the phenyl ring of the *N*-phenylpiperazine fragment. In the present study, we explore the photophysical properties of these dyes under different conditions and evaluate their interactions with double-stranded DNA (dsDNA) to assess their potential as fluorogenic probes for biomolecular recognition. This work presents a combined experimental and theoretical investigation of the optical properties—specifically UV/VIS absorption and fluorescence of three styryl hemicyanine dyes with benzo[*d*]thiazolium and substituted *N*-phenylpiperazine end groups. The primary aim is to understand the influence of substituents on the dyes’ optical behavior and to assess the extent of intramolecular charge transfer (ICT). Density functional theory (DFT) and time-dependent DFT (TDDFT) calculations were employed to evaluate key electronic characteristics, including HOMO–LUMO energy levels, absorption, and emission spectra.

## 2. Results and Discussion

### 2.1. Synthesis of Target Molecules

**Scheme 1:**
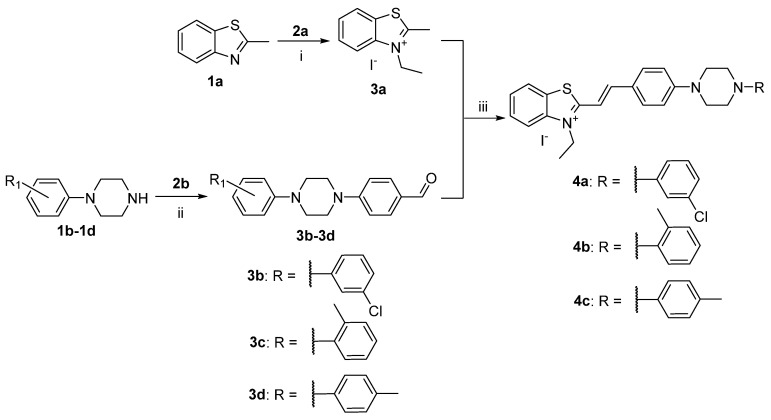
Synthesis of hemicyanine dyes with benzo[*d*]thiazolium and *N*-phenylpiperazine end groups. Reagents and conditions: (i) **1a** and **2a**, sealed tube, 110 °C; (ii) K_2_CO_3_, NMP, 145 °C, 8 h; (iii) dry ethanol, boiling temperature, DIPEA, reflux, inert atmosphere, absence of light, 3 h; yields: **4a** 76%, **4b** 81%, **4c** 70%.

For the synthesis of the styryl hemicyanine dyes **4a**, **4b**, and **4c** from the series, we used the reaction pathway represented in Scheme 1. In a previous work, we demonstrated a time-efficient chemical approach for quaternization of benzothiazole derivatives [14]. Using an environmentally friendly procedure, we carried out the quaternization reaction between 2-methylbenzothiazole (**1a**) and iodoethane (**2a**) in a sealed tube, under pressure, at 110 °C. As a result of this nucleophilic substitution reaction, 3-ethyl-2-methylbenzothiazolium iodide (**3a**) was obtained in excellent yields and purity (TLC). The reaction of aromatic nucleophilic substitution of fluorine atom, in the presence of potassium carbonate as a base, and in the high-boiling NMP as a solvent, between the substituted *N*-phenylpiperazines (**1b**–**1d**) and 4-fluorobenzadehyde (**2b**) afforded a series of 4-*N*-phenylpiperazine-benzaldehydes (**3b**–**3d**) (Scheme 1). The condensation reaction between the quaternary benzothiazolium salt **3a** and the corresponding aldehydes **3b**, **3c**, or **3d** afforded the monocationic styryl dyes **4a**–**4c** in excellent yields. The reagents were ground together in a mortar and suspended in dry ethanol, in the presence of catalytic amounts of *N, N*-diisopropylethylamine (DIPEA) as a basic reagent. After boiling for three hours and cooling the reaction mixture, the precipitated dyes were further isolated by filtration and dried using a vacuum dryer. The improvement in the procedure was related to the reduced amount of solvent, which permitted the direct crystallization of the target dyes with no need for further recrystallization. Another improvement is the use of sterically hindered Hünig’s base DIPEA instead of the conventional piperidine. DIPEA is capable to deprotonate only the methyl group on the second position in the benzothiazole ring. The use of a 20% molar excess of the well-soluble in ethanol *N*-phenylpiperazine-benzaldehyde leads to complete reaction of the 3-ethyl-2-methylbenzothiazolium salt and direct precipitation of a pure product, without the need for additional purification. The only impurity we noticed was ethanol (ECI), which, if necessary, can be reduced after heating to 50 °C in a vacuum oven for 48 h. Regarding the purity of the products, it can be said that it is over 99%, obtained directly from the reaction mixture without additional purification. The chemical structures were confirmed by TLC and then by NMR spectroscopy, with a high purity (^1^H-NMR, ^13^C-NMR, and HMBC are given in the Supplementary Materials).

### 2.2. Photophysical Properties of Dyes ***4a***–***4c*** in Solution and in the Presence of HCl and DNA

#### 2.2.1. Absorption

**Figure 1:**
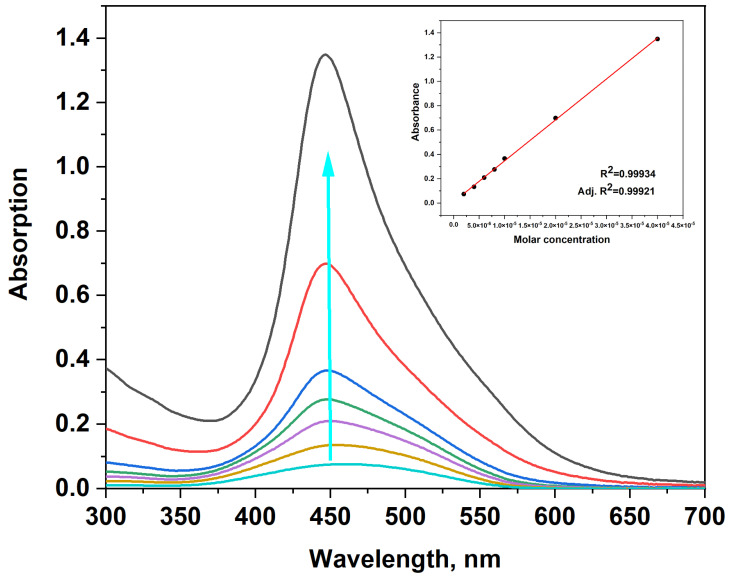
UV/VIS spectra of dye **4a**, c = (0.2; 0.4; 0.6; 0.8; 1; 2; 4) × 10^−5^ M in TE buffer; the blue arrow indicates the increasing dye concentration. Inset: calibration curve showing absorbance as a function of dye concentration.

The main photophysical properties of the novel dyes were characterized using UV/VIS and fluorescence spectroscopy. The absorption spectra of the dyes were carried out in TE buffer with pH = 8 (10 mM Tris-HCl, 1 mM EDTA) (Figure 1). The dyes exhibit a single absorption band in the visible region, ruling out the possibility of aggregate formation under the experimental conditions. Dye **4a** displayed an absorption maximum at 449 nm (Figure 1), while dyes **4b** and **4c** showed maxima at 460 nm (Figures S1–S3, Supplementary Materials). The wavelengths corresponding to the maximum absorption (λ_abs_) and molar absorptivity (ε) are summarized in Table 1.

**Figure 2:**
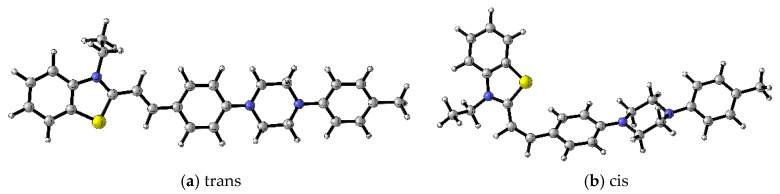
B3LYP/6-31+G(d,p) optimized geometries of *cis* and *trans* isomers of **4c**. Color scheme: C—gray, S—yellow, H—white, N—blue [17].

**Figure 3:**
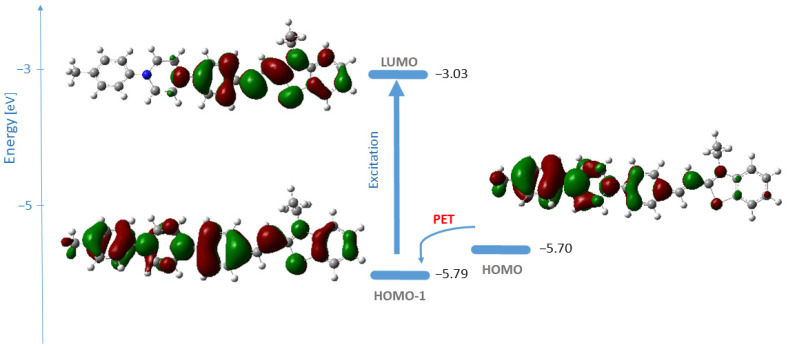
Energies and shape representation of HOMO-1, HOMO, and LUMO for the excited state of **4c** in water from B3LYP/6-31+G(d,p) computations. The vertical arrow represents the electronic transition; the curved arrow indicates the possibility for photoinduced electron transfer [18,19].

The character of the transitions and the influence of the substituents on the spectral properties were studied with DFT/TDDFT computations. The geometry optimization and photophysical properties of the dyes were computed using the G16 software package [16]. Various isomers for **4a**, **4b**, and **4c** were optimized at B3LYP/6-31+G(d,p) level of theory in dichloromethane solvent. One trans and one cis conformer were considered (Figure 2). The trans isomer was found to be the most thermodynamically stable. The relative energy of the cis isomer was calculated to be 8.1 kcal/mol higher than the trans isomer. These results confirm a strong preference for the trans geometry under equilibrium conditions. From now on, only the properties of the stable trans isomers will be discussed (Figure S4). The optical responses of the studied dyes in water and toluene were simulated using TDDFT computations employing the PBE0 functional in conjunction with 6-311+G(d,p) basis set. The computed transition energies and the respective oscillator strength (*f*), summarized in Table 2, complement the experimental results for the dyes **4a**–**4c**. The energies and shape representation of the frontier molecular orbitals for optimized S_0_ state geometry from TDB3LYP/6-31+G(d,p) computations are shown in Figure 3.

Theoretical computations in water revealed that the orbitals involved in the vertical transition of the fluorophore, and responsible for the band observed in the spectra, are HOMO-1 → LUMO (λ_comp_ = 469 nm, oscillator strength 1.2540) (Table 2, Figure 3). The HOMO → LUMO transition was calculated at 486 nm with an oscillator strength of 0.5439 (Table 2). This transition has charge transfer (CT) character as seen from Figure 3. In the experimental UV/VIS spectra of **4c** (Figure 1), the band associated with the HOMO-LUMO transition is likely hidden beneath the more intense absorption band due to the HOMO-1 → LUMO transition. The HOMO-1 of the **4c** ground state is delocalized over the whole molecule, while LUMO covers the acceptor part. The orbitals’ shape indicates that an effective intramolecular charge transfer occurs from the donor group (*N*-phenylpiperazine substituent) toward the benzothiazole moiety. Hence, the HOMO-1 → LUMO transition is classified as an internal charge transfer (ICT) transition. As can be seen from Figure 3, the electron density of HOMO is distributed over the *N*-phenylpiperazine moiety, the receptor, whereas HOMO-1 represents the fluorophore. The electron density of LUMO is centered on the hemicyanine part—the fluorophore. The lone pair orbitals of the amino groups in the receptor are of higher energies than those of the fluorophore, which is a precondition for a typical reductive photoinduced electron transfer process. Thus, upon excitation, an electron is transferred from the receptor to the fluorophore, resulting in the quenching of **4c** emission [18,19].

**Figure 4:**
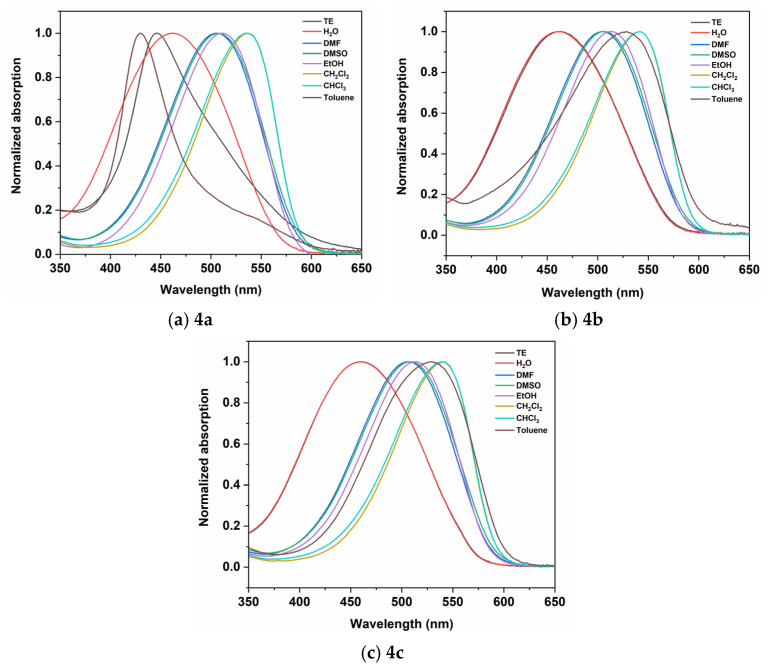
Normalized spectra of **4a**–**4c** in solvents with different polarity. The concentration of the dyes is 2 × 10^−5^ M.

As mentioned, the theoretical calculations revealed that the longest-wavelength absorption corresponds to an intramolecular charge transfer (ICT) transition (Figure 3). The nature of this ICT transition implies a strong sensitivity to solvent polarity, suggesting that the position of the charge transfer (CT) band would be significantly influenced by the surrounding medium. To investigate the solvatochromic behavior of the dyes in detail, absorption spectra were recorded in solvents of varying polarity—H_2_O, DMF, DMSO, EtOH, CH_2_Cl_2_, CHCl_3_, and toluene. Solvatochromism is the phenomenon where the position and/or the intensity of the molecular electronic absorption or emission spectrum changes with the nature or polarity of the solvent [20]. This spectral shift occurs due to the different stabilization of the molecule’s ground and excited states by the surrounding media. The studied dyes are highly solvatochromic, showing both horizontal and vertical solvatochromism (Figure 4 and Figures S5–S7). The normalized spectra of dyes **4a**, **4b**, and **4c** in various solvents are shown in Figure 4.

All dyes exhibit negative solvatochromism when going from nonpolar to polar solvents (e.g., from toluene to DMSO). Negative solvatochromism is characterized by a hypsochromic (blue) shift in the absorption maximum with increasing solvent polarity, indicating that the ground state is more polar than the excited state. For example, dye **4a** shows a significant shift of 3951 cm^−1^, from λ_max_ = 537 nm in CH_2_Cl_2_ to λ_max_ = 443 nm in TE buffer. The shift for **4b** and **4c** is 3255 cm^−1^ and 3221 cm^−1^, respectively, going from CHCl_3_ and CH_2_Cl_2_ to H_2_O solvent. The absorption maxima of the dyes in different solvents are summarized in Table 3.

The Kamlet–Taft equation (Equation (1)) was used to quantify the solvatochromic behavior of the dyes:

(1) $$\lambda_{max}=y_{0}+a\alpha +b\beta +{c\pi }^{*}$$

In Equation (1), *y*_0_ is the absorption of a reference solvent such as cyclohexane. The variables *a*, *b*, and *c* are adjustable coefficients that determine the contribution of the corresponding Kamlet–Taft (KT) parameters (*α*, *β*, π*) to λ_max_ [21,22,23,24] (Table S1, Supplementary Materials). The KT scale considers the acidity, basicity, and dipolarity/polarisability of the solvents. π* measures the ability of the solvent to stabilize a charge or a dipole through its dielectric constant. The parameter α describes the ability of the solvent to donate a proton in a solvent-to-solute hydrogen bond. The *β* scale quantifies the ability of a solvent to donate an electron pair in a solute-to-solvent hydrogen bond.

The results of the multilinear regression analysis for dyes **4a**–**4c** are given in Equations (2)–(4) below.

(2) $$\begin{array}{c} \lambda_{max}=597-34.57\alpha -27.07\beta -73.7\pi^{*\;}\\ R^{2}=0.95,\;R_{adj}^{2}=0.87,\;n=6,\;F=12.8 \end{array}$$

For dyes **4a**–**4c**, the fit is good (R^2^ *≈* 0.95), indicating that the Kamlet–Taft model explains almost all of the solvent-dependent variation in λ_max_. Negative coefficients for *α*, *β*, and π* suggest that increasing solvent polarity or hydrogen-bonding ability leads to a blue shift. For all three dyes, toluene is an outlier that can be due to stacking *π*–*π* interaction between the solvent molecules and the dye heterocycle or the formation of H-aggregates stabilized by the solvent. The model is statistically significant even with limited data. Solvents with higher polarity (π*) lead to hypochromic shift in the absorption maxima (blue shifts). This could mean that the excited state is less polar than the ground state, and polarity stabilizes the ground state more. Hydrogen bond donor solvents also cause a blue shift, and hydrogen bond formation stabilizes the ground state more strongly. *β* indicates that solvents with hydrogen bond acceptor properties also induce a blue shift in the absorption, although the effect is weaker. The Kamlet–Taft analysis confirms that dipolarity (π*) contributes the most. The strong negative c coefficient indicates that solvent polarity is the dominant factor—polar solvents raise the transition energy (shorter λ_max_). The coefficients for α and β show that both hydrogen-bond donor and acceptor interactions stabilize the ground state relative to the excited state. Thus, as solvent polarity and hydrogen bonding ability increase, the absorption maximum blue-shifts. This is consistent with n → π* or charge–transfer transitions, where the excited state is more delocalized and less stabilized by polar environments.

(3) $$\begin{array}{c} \lambda_{max}=609-35.92\alpha -34.33\beta -80.91\pi^{*}\\ R^{2}=0.96,\;R_{adj}^{2}=0.90,\;n=6,\;F=15.77 \end{array}$$

(4) $$\begin{array}{c} \lambda_{max}=608-36.66\alpha -31.62\beta -80.52\pi^{*}\\ R^{2}=0.96,\;R_{adj}^{2}=0.89,\;n=6,\;F=15.77 \end{array}$$

**Figure 5:**
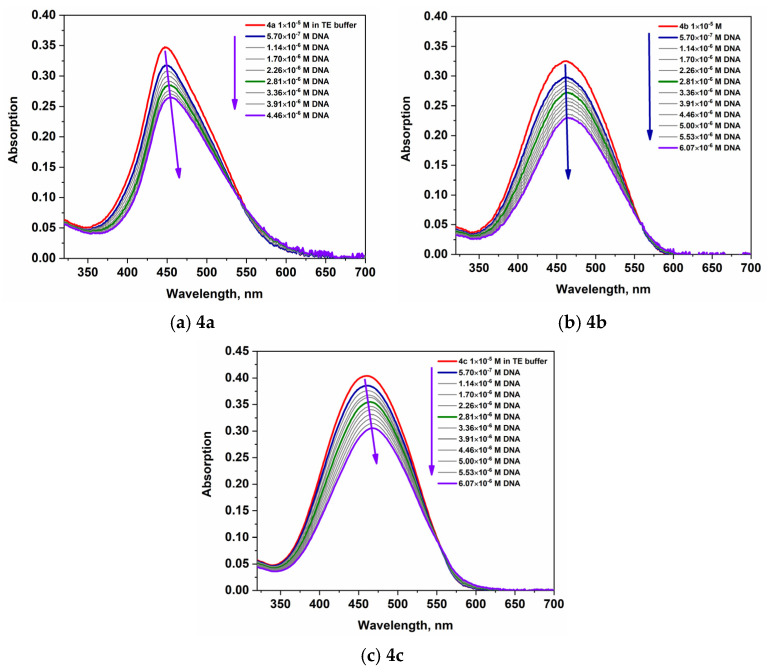
Absorption spectra of **4a**–**4c** upon titration with DNA.

**Figure 6:**
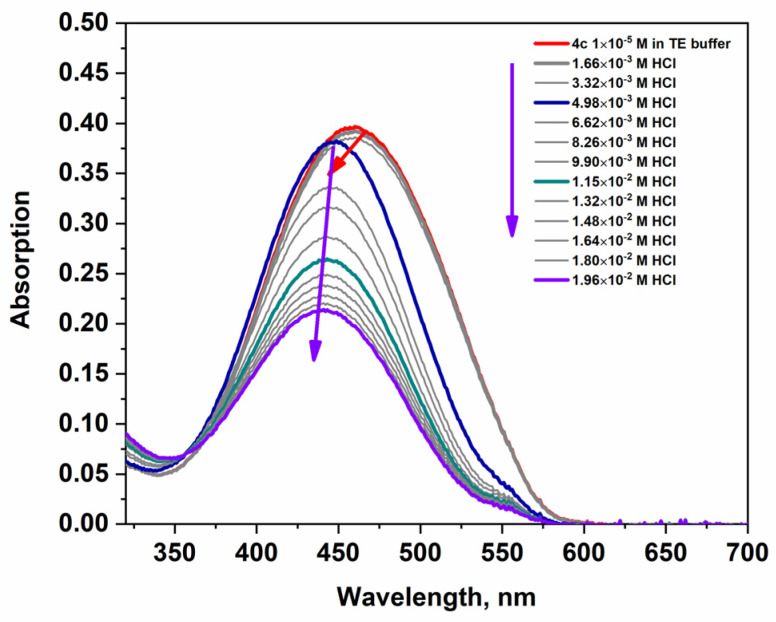
The changes in the absorption spectra of **4c** upon titration with HCl. The violet arrows indicate the decrease in intensity of the band, and the red arrow indicates the hypsochromic shift in the absorption band after the addition of acid.

Changes in the absorption and fluorescence spectra of the dyes were studied in the presence of DNA and HCl to understand how these interactions affect their spectral characteristics compared to the free (unbound) state. The spectral characteristics of the studied dyes in buffer solution and in the presence of DNA and HCl are summarized in Table 1, Figures S10 and S11, in the Supplementary Materials. The data on the molar absorbance of the longest wavelength absorption band, presented in Table 1, show a significant decrease in the molar absorbance upon addition of hydrochloric acid and upon maximum saturation with DNA. Compounds **4a**–**4c** were evaluated for their binding affinity to dsDNA. The addition of DNA to the dyes leads to decreasing absorption of the most intense band in the visible spectrum and a bathochromic shift in its position (+8 nm for **4a**, **4b,** and **4c**), demonstrated in Figure 5. This is evidence of an interaction between the NA and the dyes and may be attributed to a groove-binding mode. The spectral behavior of the dyes upon addition of HCl is shown in Figure 6, Figures S8 and S9. It can be seen, from Figure 6 and the data presented in Table 1, that when titrating a buffer solution of the dyes with a hydrochloric acid solution, the molar absorbance decreases by almost one-third of the initial value. Dyes **4b** and **4c** exhibit pronounced acidochromism; in particular, the addition of HCl to dye 4c induces a hypochromic effect accompanied by a hypsochromic shift of up to 20 nm in the absorption maximum (Figure 6).

**Figure 7:**
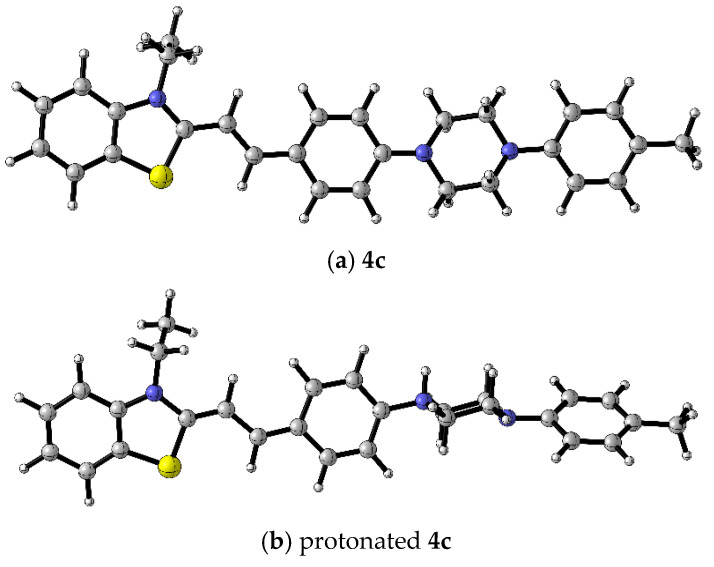
B3LYP/6-31+G(d,p) optimized geometries of (**a**) **4c** and (**b**) protonated form of dye **4c**. Color scheme: C—gray, S—yellow, H—white, N—blue [17].

The geometry of the protonated dye **4c** was optimized with quantum chemical computations. The results show that the protonated product at the first N atom is energetically more stable. The protonation of the first nitrogen atom leads to a distortion in the planarity of the molecule (Figure 7).

The computed transition energy and oscillator strength (*f*) results for dye **4c**, summarized in Table 4, complement the experimental results.

The good agreement between the theoretical and experimental spectral characteristics provides additional evidence that the chosen functional and basis set are suitable for this class of chromophores. The observed hypsochromic shift in the absorption maximum of the protonated dye **4c** can be attributed to structural changes induced by protonation, specifically a reduction in the *π*-conjugation system.

#### 2.2.2. Fluorescence

The fluorescence properties of dyes **4a**–**4c** were studied under four conditions: the dye only, in the presence of hydrochloric acid, in the presence of dsDNA, and in the presence of both HCl and DNA. The dyes exhibit low intrinsic fluorescence in solution with maxima at λ_em_ = 590 nm for **4a**, λ_em_ = 589 nm for **4b**, and λ_em_ = 587 nm for **4c** (Table 5). The data in Table 5 clearly demonstrate the unique properties of the obtained new dyes **4a**–**4c**. It can be seen that the highest values of fluorescence intensity are observed in acidic solutions and in those with the co-presence of DNA and acid. An additional advantage of these dyes is the large Stokes shifts of the fluorescent bands, which is a sought-after advantage in a number of bioanalytical techniques. The fluorescence maxima are bathochromically shifted relative to the absorption maxima, leading to large Stokes shifts of about 4703 cm^−1^ for **4c**.

**Figure 8:**
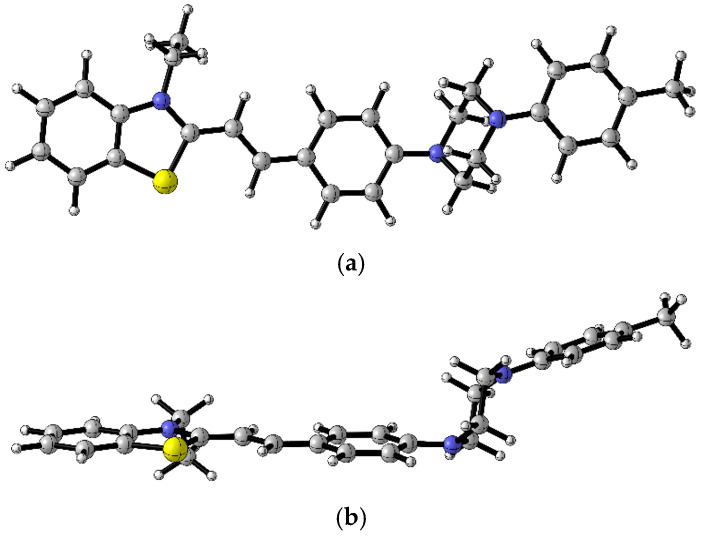
B3LYP/6-31+G(d,p) optimized geometry of the first excited state (S_1_) of 4c (**a**) top view, (**b**) side view. Color scheme: C—gray, S—yellow, H—white, N—blue [17].

This significant shift suggests that substantial conformational changes occur during the relaxation from the Franck–Condon state to the locally excited (LE) emissive state. With the help of TDDFT, the geometry of the excited state (S_1_) for dye **4c** was optimized (Figure 8). The geometry of the excited state is distorted, and the molecule is no longer planar.

**Figure 9:**
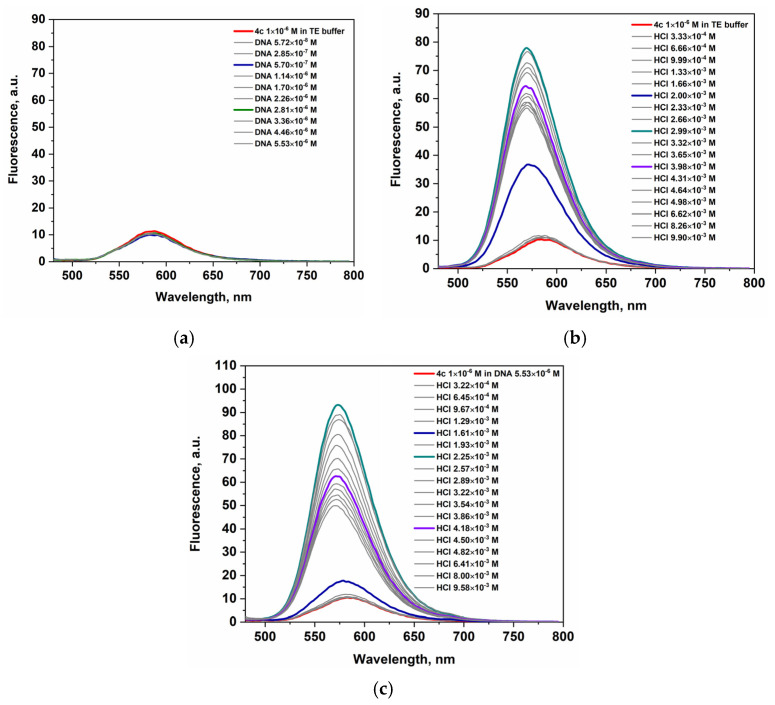
Emission spectra of (**a**) **4c** upon titration with DNA; (**b**) **4c** upon titration with HCl; (**c**) **4c**-dye-DNA complex upon titration with HCl.

Upon addition of dsDNA, all three dyes show fluorescence quenching (Figure 9a, Figures S12A and S13A). The addition of HCl to the dye’s solution leads to a noticeable increase in fluorescence intensity, with compound **4a** reaching its maximum at 2.12 × 10^−2^ M HCl (Figure S12B). Compounds **4b** and **4c** display similar behavior, achieving peak emission at lower HCl concentrations—3.21 × 10^−3^ M for **4b** and 2.89 × 10^−3^ M for **4c** (Figure 9b and Figure S13B). Beyond these concentrations, fluorescence intensity begins to decline in all cases. The fluorescence quenching is attributed to the PET effect from the nitrogen atoms of the piperazine substituent, as demonstrated by theoretical computations. The titration with hydrochloric acid enhances fluorescence of dyes in solution and their respective dye–DNA complexes, providing further evidence for the intramolecular PET effect. The emission maximum of the dye–DNA complexes in the presence of HCl is hipsochromically shifted (20 nm for dye **4c**) compared to the λ_em_ of the pure dye. Upon titration of the dye–DNA complexes with HCl, the fluorescence intensity increases, reaching a maximum at 2.51 × 10^−2^ M HCl for compound **4a** (Figure S12C), and at 3.21 × 10^−3^ M and 2.89 × 10^−3^ M for compounds **4b** and **4c**, respectively (Figure 9c and Figure S13C).

The titration of **4c**-dye–DNA complex (Figure 9c) with HCl solution results in a stronger fluorescence response compared to dye **4c** in the presence of acid (Figure 9b). Among the three dyes studied, the **4c**-dye-DNA complex exhibits the most pronounced fluorescence enhancement in acidic conditions. The apparent average values of the increase in fluorescence intensities (Table 5) upon titration with hydrochloric acid of the dye-DNA complexes only partially characterize the fluorescence responses. The amount of emitted fluorescence is fully sufficient for bioanalytical purposes. In addition, we can emphasize that the fluorogenicity and operability of the dyes in the presence of both DNA and acids make them very promising markers in the study of malignant cell lines.

### 2.3. Molecular Docking Analysis

**Figure 10:**
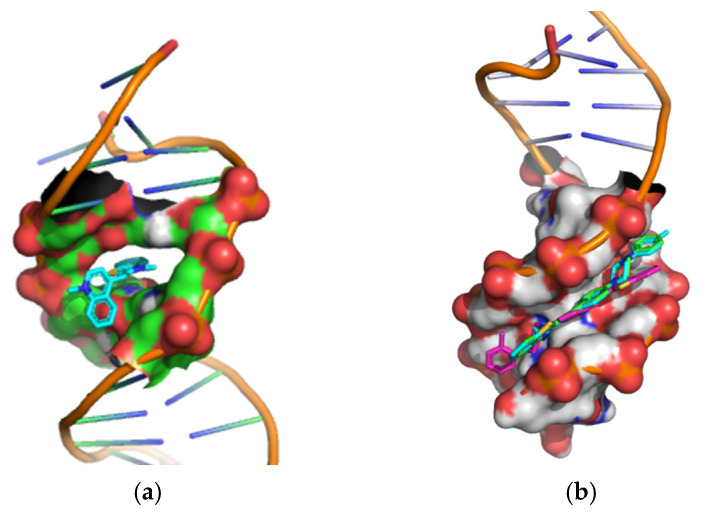
Cartoon-surface representation of dsDNA (PDB: 1G3X) in a complex with (**a**) thiazole orange; (**b**) styryl dyes **4a** (green), **4b** (magenta), **4c** (cyan).

Molecular docking experiments were conducted to investigate the interactions between the examined styryl dyes and dsDNA. The 3D XRD structure of 1G3X, which represents dsDNA with an intercalated ligand, was used as the docking template. In order to validate our docking procedure, thiazole orange (TO), a well-characterized intercalating agent, was used in a comparative study. As expected, TO bound in an intercalative mode, confirming the reliability of our docking approach (Figure 10a).

In the docking procedure, the geometries of the dyes’ structures (optimized by DFT computations) were treated as flexible, while the molecule of the dsDNA was kept rigid. This approach enabled us to identify the binding modes of the most energetically favorable ligand–receptor complexes. The binding energies of the nine conformers with the lowest predicted binding affinities are given in Table S2. The results indicate that each of the ligands **4a**–**4c** interacts with dsDNA through hydrophobic interactions within the minor groove. The most favorable binding poses for the complexes of dyes **4a**–**4c** and TO with dsDNA (1G3X) are shown in Figure 10, and their respective interaction energies are listed in Table 6. The calculated interaction energies support the conclusion that the minor groove is the preferred binding site for the new ligands, whereas TO exhibits an intercalative binding mode.

### 2.4. Circular Dichromism Measurements

**Figure 11:**
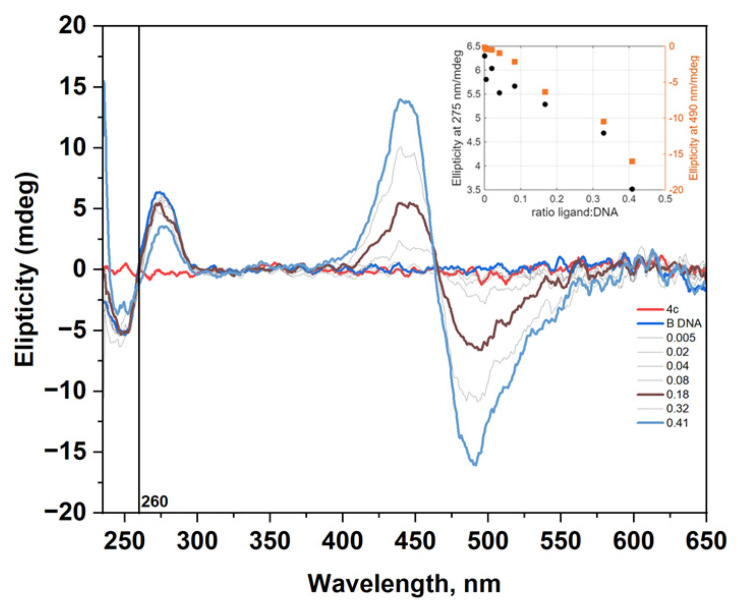
The changes in the CD spectrum of B-DNA (c = 1.1 × 10^−4^ mol/L) in TE buffer (10 mM Tris-HCl, 1mM EDTA, pH = 7.5) upon addition of dye **4c** at different ratios r = [dye]/[polynucleotide].

It was important to establish whether the dyes indeed interact with nucleic acids. To verify this, we examined the interaction of dye **4c** with B-DNA using circular dichroism (CD) spectroscopy, a valuable method for studying the binding of small molecules to chiral macromolecules such as nucleic acids (NA). Circular dichroism titration of dye **4c** with B-DNA was performed. The CD spectra shown in Figure 11 provide clear evidence of complex formation, even though the fluorescence spectra of the **4c**–DNA complexes (Figure 9a) show little to no change. During the titration experiment, the nucleotide concentration was kept constant while the dye was gradually added to the NAs solution. The CD spectra of dye **4c** interacting with dsDNA are presented in Figure 11. The CD spectrum of the B-DNA structure at pH 7.5 has a positive band around 275 nm and a negative band around 249 nm. Dye **4c** itself does not show any characteristic CD in its absorption interval. The induced CD signal (ICD) observed in the absorption band of a nonchiral ligand is clear evidence of interaction with NA. The appearance of exciton-coupled bisignate ICD bands is evidence of the dye interacting with the B-DNA. The form of the ICD strongly supports binding along the groove of the polynucleotides or aggregation in the groove of the NA.

The CD spectra recorded during the titration of B-DNA with dye **4c** (Figure 11) were analyzed using multivariate data analysis to explore the possible formation of multiple interaction complexes between the dye and nucleic acid [25]. The method allowed the calculation of the concentration profiles and pure spectra for the considered components (Figure S14). In this case, only two components were detected, corresponding to free DNA and the dye–DNA complex.

## 3. Materials and Methods

The starting materials **1a**, **2a**, **1b**–**1d**, and **2b,** and all the solvents used in our research are HPLC grade and commercially available. Melting points of dyes **4a**–**4c** were determined on an automated melting point apparatus SRS EZ-Melt MPA120 (Stanford Research Systems, Sunnyvale, CA, USA) at a ramp rate of 1 °C/min and are presented uncorrected. NMR spectra were obtained on a Bruker Avance II + NMR spectrometer operating at 500 MHz for ^1^H-NMR and 125 MHz for ^13^C-NMR. The chemical shifts are given in parts per million for the spectra in DMSO-*d*_6_ relative to the solvent residual peak [26]. The nature of electronic transitions and their manifestation in the electronic spectra were revealed by DFT and TDDFT calculations performed with the G16 software package [16].

### 3.1. Synthesis of Dyes ***4a***, ***4b***, ***4c***

To obtain dyes **4a**, **4b,** and **4c**, the reactions between the quaternary benzothiazolium salt **3a** (0.5 mmol, 0.152 g) and the corresponding aldehyde **3b**, **3c**, or **3d**, respectively (0.6 mmol, 0.180 g for **3b**, 0.168 g for **3c** and **3d**) were performed under reflux, in the presence of absolute ethanol (10 mL) and 2 drops of DIPEA. Precipitation of the products was observed during the first hour of the synthesis. The progress of the reaction was followed by TLC (petroleum ether/ethyl acetate 4.5:0.5), and, after 3 h of being vigorously stirred at the boiling temperature of the solvent, under an inert atmosphere, the reaction was considered finished. The formed precipitates were left overnight in the fridge, then suction filtered, washed with small amounts of cold ethanol, and dried in a vacuum oven at 50 °C for 48 h.

(*E*)-2-(4-(4-(3-chlorophenyl)piperazin-1-yl)styryl)-3-ethylbenzo[*d*]thiazol-3-ium iodide (**4a**): (Yield 0.223 g, 76%). Mp 262.1–266.5 °C. ^1^H-NMR (DMSO-d_6_, δ (ppm)): 1.44 (3H, t, H-1, J = 7.1 Hz); 3.37 (4H, t, H-17, H-17`, J = 5.0 Hz); 3.62 (4H, t, H-16, H-16`, J = 5.1 Hz); 4.88 (2H, q, H-2, J = 7.1 Hz); 6.82 (1H, dd, H-21, J = 1.5 Hz, J = 7.8 Hz); 6.96 (1H, dd, H-19, J = 2.2 Hz, J = 8.4 Hz); 7.00 (1H, t, H-23, J = 2.0 Hz); 7.13 (2H, d, H-14, H-14`, J = 9.0 Hz); 7.25 (1H, t, H-20, J = 8.1 Hz); 7.70–7.74 (2H, m, H-5, J = 7.7 Hz, H-11, J = 15.3 Hz); 7.81 (1H, dt, H-6, J = 1.0 Hz, J = 7.9 Hz); 7.98 (2H, d, H-13, H-13`, J = 8.9 Hz); 8.12 (1H, d, H-10, J = 15.3 Hz); 8.19 (1H, d, H-7, J = 8.5 Hz); 8.35 (1H, d, H-4, J = 8.0 Hz). ^13^C-NMR (DMSO-d_6_, δ (ppm)): 13.97 (1C, C-1); 43.78 (1C, C-2); 45.92 (2C, C-16, C-16`); 47.14 (2C, C-17, C-17`); 107.31 (1C, C-11); 113.67 (1C, C-10); 113.70 (2C, C-14, C-14`); 114.54 (1C; C-23); 115.97 (1C, C-7); 118.26 (1C, C-21); 123.27 (1C, C-12); 124.13 (1C, C-4); 127.48 (1C, C-5); 127.71 (1C, C-8); 129.14 (1C, C-6); 130.51 (1C, C-20); 132.72 (2C, C-13, C-13`); 133.87 (1C, C-18); 140.86 (1C, C-3); 150.12 (1C, C-10); 151.78 (1C, C-22); 153.39 (1C, C-15); 171.21 (1C, C-9).

(*E*)-3-ethyl-2-(4-(4-(o-tolyl)piperazin-1-yl)styryl)benzo[*d*]thiazol-3-ium iodide (**4b**): (Yield 0.252 g, 81%). Mp 299.2–300.7 °C. ^1^H-NMR (DMSO-d_6_, δ (ppm)): 1.45 (3H, t, H-1, J = 7.2 Hz); 2.32 (3H, s, H-24); 3.00 (4H, t, H-17, H-17`, J = 4.5 Hz); 3.62 (4H, t, H-16, H-16`, J = 4.4 Hz); 4.88 (2H, q, H-2, J = 7.0 Hz); 6.99 (1H, t, H-21, J = 7.4 Hz); 7.07 (1H, d, H-23, J = 7.8 Hz); 7.14–7.21 (4H, m, H-14, H-14`, J = 9.0 Hz, H-20, H-22); 7.70–7.75 (2H, m, H-11, J = 15.6 Hz; H-5, J = 7.6 Hz); 7.81 (1H, t, H-6, J = 7.7 Hz); 7.98 (2H, d, H-13, H-13`, J = 8.8 Hz); 8.13 (1H, d, H-10, J = 15.4 Hz); 8.19 (1H, d, H-7, J = 8.4 Hz); 8.35 (1H, d, H-4, J = 8.0 Hz). ^13^C-NMR (DMSO-d_6_, δ (ppm)): 13.97 (1C, C-1); 17.60 (1C, C-24); 43.78 (1C, C-2); 46.90 (2C, C-16, C-16`); 51.14 (2C, C-17, C-17`); 107.37 (1C, C-11); 113,81 (2C, C-14, C-14`); 115.98 (1C, C-7); 118.88 (1C, C-23); 123.23 (1C, C-12); 123.33 (1C, C-21); 124.14 (1C, C-4); 126.64 (1C, C-20); 127.50 (1C, C-5); 127.73 (1C, C-8); 129.15 (1C, C-6); 130.92 (1C, C-22); 131.86 (1C, C-19); 132.71 (2C, C-13, C-13`); 140.88 (1C, C-3); 150.13 (1C, C-10); 150.84 (1C, C-18); 153.79 (1C, C-15); 171.25 (1C, C-9).

(*E*)-3-ethyl-2-(4-(4-(p-tolyl)piperazin-1-yl)styryl)benzo[*d*]thiazol-3-ium iodide (**4c**): (Yield 0.198 g, 70%). Mp > 300 °C. ^1^H-NMR (DMSO-d_6_, δ (ppm)): 1.44 (3H, t, H-1, J = 7.2 Hz); 2.21 (3H, s, H-22); 3.23 (4H, t, H-17, H-17`, J = 5.1 Hz); 3.62 (4H, t, H-16, H-16`, J = 5.1 Hz); 4.88 (2H, q, H-2, J = 7.2 Hz); 6.91 (2H, d, H-19, H-19`, J = 8.5 Hz); 7.06 (2H, d, H-20, H-20`, J = 8.5 Hz); 7.13 (2H, d, H-14, H-14`, J = 9.0 Hz); 7.70–7.74 (2H, m, H-11, J = 15.0 Hz, H-5, J = 7.7 Hz); 7.8 (1H, dt, H-6, J = 7.9 Hz, J = 0.8 Hz); 7.98 (2H, d, H-13, H-13`, J = 9.0 Hz); 8.12 (1H, d, H-10, J = 15.3 Hz); 8.19 (1H, d, H-7, J = 8.4 Hz); 8.35 (1H, d, H-4, J = 8.0 Hz). ^13^C-NMR (DMSO-d_6_, δ (ppm)): 13.97 (1C, C-1); 20.06 (1C, C-22); 43.77 (1C, C-2); 46.24 (2C, C-16, C-16`); 48.48 (2C, C-17, C-17`); 107.29 (1C, C-11); 113.76 (2C, C-14, C-14`); 115.93 (2C, C-19, C-19`); 115.96 (1C, C-7); 123.26 (1C, C-12); 124.12 (1C, C-4); 127.47 (1C, C-5); 127.70 (1C, C-8); 128.13 (1C, C-21); 129.13 (1C, C-6); 129.45 (2C, C-20, C-20`); 132.72 (2C, C-13, C-13`); 140.86 (1C, C-3); 148.58 (1C, C-18); 150.12 (1C, C-10); 153.53 (1C, C-15); 171.19 (1C, C-9).

### 3.2. Photophysical Properties of Dyes ***4a***, ***4b***, ***4c***

UV/VIS absorption spectra were recorded using a Shimadzu UV-1800 spectrophotometer, while fluorescence spectra were measured on a Perkin Elmer LS45 fluorescence spectrophotometer (slit widths are 5 nm, in a quartz cuvette—10 × 10 mm). Stock solutions of the dyes were prepared in DMSO with a concentration of 2 × 10^−3^ M, and further diluted with TE-buffer (Tris-HCl 10 mM, pH 8.0; EDTA 1 mM, pH 8.0). The absorption and emission properties of dyes **4a**–**4c** were investigated neat in TE buffer and in the presence of dsDNA. The dyes’ working concentrations were 1 × 10^−5^ M for the absorption and 1 × 10^−6^ M for the fluorescence measurements. All UV/VIS and fluorimetric titrations were carried out at room temperature by keeping the dye concentration constant while varying the DNA and HCl concentration. The working DNA concentration was 3 × 10^−5^ M. DNA purity and concentration were confirmed spectroscopically at 260 and 280 nm using the extinction coefficient of dsDNA (ε = 6600 M^−1^·cm^−1^) [27]. During titrations, a 1M HCl solution was used as the acid source. For solvatochromic studies, dye solutions were prepared by diluting an aliquot of the 2 mM stock solution to a final concentration of 2 × 10^−5^ M using solvents of varying polarity, including H_2_O, DMF, DMSO, EtOH, CH_2_Cl_2_, CHCl_3_, and toluene. CD spectra of dye **4c** were recorded on a JASCO J-815 spectrophotometer from 230 to 650 nm at room temperature. A quartz cuvette cell (10 × 10 mm) was used. A 10 L⋅min^−1^ N2 flow was used during the measurements. CD experiments were performed by adding portions of **4c** dye into the solution of the dsDNA (c = 1 × 10^−4^ M). After each addition, spectra were measured as the average of two recordings with a speed rate of 100 nm·min^−1^. The CD spectrum of the blank was subtracted accordingly.

### 3.3. Molecular Docking of Dyes ***4a***, ***4b***, ***4c***

For the molecular docking, we used the Autodock 4.2.6 suite of programs (The Scripps Research Institute, La Jolla, CA, USA). The preparation of the pdbqt files, as well as the determination of the grid box size, were carried out using Autodock Tools (ADT) version 1.5.6. The docking results were visualized in 3D using the PyMOL (version 2.5.2) molecular graphics system. The crystallographic structure of the receptor was downloaded from the protein databank with the following code: DNA (PDB: 1G3X).

## 4. Conclusions

An improved synthetic strategy was developed for a series of styryl hemicyanine dyes bearing benzo[*d*]thiazolium and substituted *N*-phenylpiperazine end groups. Utilizing a sterically hindered base in combination with minimal solvent resulted in high-purity products with yields exceeding 70%. The dyes exhibit pronounced negative solvatochromism, as well as acidochromic behavior. The dyes display weak intrinsic fluorescence in solution, which is quenched upon interaction with DNA. Fluorescence intensity increases in the simultaneous presence of both DNA and H^+^ ions. Spectroscopic titrations (UV/VIS and fluorescence) with DNA and HCl, supported by DFT/TDDFT calculations, confirm the presence of an intramolecular photoinduced electron transfer (PET) effect, involving electron transfer from the *N*-phenylpiperazine donor to the hemicyanine acceptor unit. Circular dichroism (CD) spectroscopy and molecular docking further reveal that the dyes interact with DNA via a minor-groove binding mode. Spectroscopic investigations “in cuvette” revealed fluorescence enhancement under acidic conditions, as well as interaction with DNA, suggesting that these dyes have the potential to serve as functional fluorescent probes for monitoring pH variations and nucleic acid dynamics in cancer cells. The obtained results represent an initial step in an ongoing investigation aimed at optimizing the PET-related properties through structural modification, with the goal of enabling their application in biological cellular environments with acidic conditions. Future studies should focus on evaluating their in vitro biocompatibility, cytotoxicity, and cellular uptake efficiency to establish their suitability for live-cell imaging and diagnostic applications.

## Figures and Tables

**Table 1:** UV/VIS absorption spectral characteristics of styryl hemicyanine dyes **4a**–**4c** in free state, in presence of HCl acid, and in DNA presence.

Entry	Dye ^1^		in HCl Presence ^2^		in DNA Presence ^3^	
	ε (M^−1^ cm^−1^)	λ_abs_	ε (M^−1^ cm^−1^)	λ_abs_	ε (M^−1^ cm^−1^)	λ_abs_
**4a**	33,652	449	27,200	449	26,500	455
**4b**	32,746	460	17,800	442	22,990	465
**4c**	39,029	460	21,400	441	30,550	468

^1^ λ_abs_: absorption maximum wavelengths. ^2^ Maximum HCl concentration in UV/VIS absorption spectra is 1.96 × 10^−2^ M. ^3^ Maximum DNA concentration in UV/VIS spectra is 6.07 × 10^−6^ M, and maximum DNA concentration in emission spectra is 6.07 × 10^−6^ M.

**Table 2:** PCM/TDPBE0/6-311+G(d,p) calculated (in water and toluene) and experimental absorption maxima (nm) and the oscillator strengths (*f*) for the dyes **4a**–**4c**.

Entry	λ_comp_ (nm)	*f*	λ_exp_ (nm)	λ_comp_ (nm)	*f*
	Water			Toluene	
**4a**	475	1.6844	447	—	—
**4b**	476	1.7013	460	487	1.6265
**4c**	486	0.5439	460	—	—
	469	1.2540	—	490	1.8043

**Table 3:** Photophysical behavior in different solvents.

Entry	λ_max_ (nm)							
	TE	H_2_O	DMF	DMSO	EtOH	CH_2_Cl_2_	CHCl_3_	Toluene
**4a**	447	462	505	507	511	537	534	430
**4b**	460	461	505	507	512	541	542	524
**4c**	460	461	506	509	513	541	541	528

**Table 4:** PCM/TDPBE0/6-311+G(d,p) calculated and experimental absorption maxima (nm) and the oscillator strengths (*f*) for dye **4c**.

Entry	λ_comp_ (nm)	*f*	λ_comp_ (eV)	λ_exp_ (nm)
	Water			TE Buffer
**4c**	486	0.5439	2.55	—
	469	1.2540	2.64	460
**4cH^+^**	524	0.0922	2.37	553
	455	0.9244	2.72	442

**Table 5:** Fluorescence excitation and emission maxima of dyes **4a**–**4c** in buffer solution, in presence of HCl, in presence of DNA, and, finally, fluorescence of both dyes **4a**–**4c** and DNA in presence of HCl.

Entry	Dye ^1^		in HCl Presence ^2^			in DNA Presence ^3^			Dye–DNA Complex in the Presence of HCl ^4^		
	λ_ex_	λ_em_	λ_ex_	λ_em_	ΔI	λ_ex_	λ_em_	ΔI	λ_ex_	λ_em_	ΔI
**4a**	450	590	450	573	9.3	445	585	0.6	445	569	6.9
**4b**	460	589	460	572	4.9	460	589	0.5	460	575	5.01
**4c**	460	587	460	569	9.5	460	583	0.9	460	573	9.5

^1^ λ_ex_, λ_em_: wavelengths of fluorescence excitation and emission maxima. ^2^ Maximum HCl concentration in the emission spectra for **4a** is 2.12 × 10^−2^ M; for **4b** is 3.65 × 10^−3^ M, for **4c** is 2.99 × 10^−3^ M. ^3^ maximum DNA concentration in emission spectra is 6.07 × 10^−6^ M. ^4^ Maximum HCl concentration in the emission spectra is 2.51 × 10^−2^ M for **4a**-dye-DNA complex, 2.89 × 10^−3^ M for **4b**-dye-DNA complex, 2.25 × 10^−3^ M for **4c**-dye-DNA complex.

**Table 6:** Interaction energies (ΔG in kcal/mol) of ligands with dsDNA.

Minor Groove-Binding			Intercalator
**4a**	**4b**	**4c**	**TO**
−9.4	−8.7	−9.2	−8.1

## Data Availability

The original contributions presented in this study are included in the article/Supplementary Materials. Further inquiries can be directed to the corresponding authors.

## Supplementary Materials

The following supporting information can be downloaded at: https://www.mdpi.com/article/10.3390/molecules30234607/s1, Figure S1: calibration curve of compound **4a**; Figure S2: calibration curve of compound **4b**; Figure S3: calibration curve of compound **4c**; Figure S4: B3LYP/6-31+G(d,p) optimized geometries of *trans* isomers of **4a**–**4c**; Figure S5: position and intensity of the CT absorption maxima for dye **4a** (2 × 10^−5^ M) in solvents with different polarity; Figure S6: position and intensity of the CT absorption maxima for dye **4b** (2 × 10^−5^ M) in solvents with different polarity; Figure S7: position and intensity of the CT absorption maxima for dye **4c** (2 × 10^−5^ M) in solvents with different polarity; Table S1. Kamlet–Taft parameters for different solvents (α, β, *π**). Figure S8: the changes in absorption spectrum of **4a** upon titration with HCl; Figure S9: the changes in absorption spectrum of **4b** upon titration with HCl; Figure S10: absorption spectra of dyes **4a**–**4c** neat and their complexes with DNA in TE buffer; Figure S11: absorption spectra of dyes **4a**–**4c** neat and in the presence of HCl; Figure S12: emission spectra of (A) **4a** upon titration with DNA, (B) **4a** upon titration with HCl, (C) **4a**-dye-DNA complex upon titration with HCl; Figure S13: emission spectra of (A) **4b** upon titration with DNA, (B) **4b** upon titration with HCl, (C) **4b**-dye-DNA complex upon titration with HCl; Table S2: binding energy of the most favorable docking modes; Figure S14: calculated concentration profiles (a) and pure CD spectra (b) from the analysis of spectra shown in Figure 11, NMR spectra of dyes **4a**–**4c**.

## Abbreviations

The following abbreviations are used in this manuscript:

MIC	Minimum Inhibitory Concentration
AChE	Acetylcholinesterase
BuChE	Butyrylcholinesterase
