# Supplementary material for: PET-Driven Fluorescence Modulation in Halochromic Styryl Hemicyanine Dyes Targeting DNA Minor Groove
Source: Molecules. 2025 Nov 30;30(23):4607. doi: 10.3390/molecules30234607 (PMC12693099; doi:10.3390/molecules30234607)
Supplement: Supplementary file 1 [file molecules-30-04607-s001.zip › molecules-3931714-supplementary -final.pdf]

Supplementary data

PET-Driven Fluorescence Modulation in Halochromic Styryl Hemicyanine Dyes Targeting DNA Minor Groove

Teodora Aleksandrova [1], Aleksandar Pashev [1, *], Sonia Ilieva [2], Raimundo Gargallo [3], Diana Cheshmedzhieva [2,*] and Aleksey Vasilev [2,4,*]

[1] Department of Chemistry and Biochemistry, Faculty of Pharmacy, Medical University Pleven, 1 St. Kliment Ohridski Street, 5800, Pleven, Bulgaria; e-mail: Teodora.Aleksandrova@mu-pleven.bg (T.A.), Aleksandar.Pashev@mu-pleve.bg (A.P.)

[2] Faculty of Chemistry and Pharmacy, Sofia University "St. Kliment Ohridski", 1 James Bourchier Ave., 1164 Sofia, Bulgaria; e-mail: silieva@chem.uni-sofia.bg (S.I.), ohtdv@chem.uni-sofia.bg (D.C.), ohtavv@chem.uni-sofia.bg (A.V.)

[3] Department of Chemical Engineering and Analytical Chemistry, University of Barcelona, Martí i Franqués 1-11, E-08028 Barcelona, Spain; e-mail: raimon_gargallo@ub.edu (R.G.)

[4] Institute of Polymers, Bulgarian Academy of Sciences, Akad. G. Bonchev St. bl 103A, 1113 Sofia, Bulgaria; e-mail: a_vassilev@polymer.bas.bg

* Correspondence: aleksandar.pashev@mu-pleven.bg (A.P.); ohtdv@chem.uni-sofia.bg (D.C.); ohtavv@chem.uni-sofia.bg or a_vassilev@polymer.bas.bg (A.V.)

Contents

**Figure S1.** UV/VIS spectra and calibration curve of compound **4a**.	S3
**Figure S2.** UV/VIS spectra and calibration curve of compound **4b**.	S3
**Figure S3.** UV/VIS spectra and calibration curve of compound **4c**.	S3
**Figure S4.** B3LYP/6-31+G(d,p) optimized geometries of trans isomers of **4a**-**4c**.	S4
**Figure S5.** Position and intensity of the CT absorption maxima for dye **4a** (2×10^{-5} M) in solvents with different polarity.	S5
**Figure S6.** Position and intensity of the CT absorption maxima for dye **4b** (2×10^{-5} M) in solvents with different polarity	S5
**Figure S7.** Position and intensity of the CT absorption maxima for dye **4c** (2×10^{-5} M) in solvents with different polarity	S5
**Table S1.** Kamlet–Taft parameters for different solvents (α, β, π^*).	S6
**Figure S8.** The changes in absorption spectrum of **4a** upon titration with HCl..	S6
**Figure S9.** The changes in absorption spectrum of **4b** upon titration with HCl..	S6
**Figure S10.** Absorption spectra of dyes **4a-4c** neat and their complexes with DNA in TE buffer.	S7
**Figure S11.** Absorption spectra of dyes **4a-4c** neat and in the presence of HCl.	S7
**Figure S12**. Emission spectra of: **A) 4a** upon titration with DNA; **B) 4a** upon titration with HCl; **C) 4a-dye-DNA** complex upon titration with HCl.	S8
**Figure S13.** Emission spectra of: **A) 4b** upon titration with DNA **B) 4b** upon titration with HCl; **C) 4b-dye-DNA complex** upon titration with HCl.	S8
**Table S2.** Binding energy of the most favorable docking modes.	S10
**Figure S14.** Deconvolution results of the CD spectra shown in Figure 11, obtained using Multivariate Curve Resolution based on Alternating Least Squares (MCR-ALS).	S10
NMR spectra of dyes **4a-4c**	S11

UV/VIS spectra and calibration curves for molar extinction coefficients (ε)

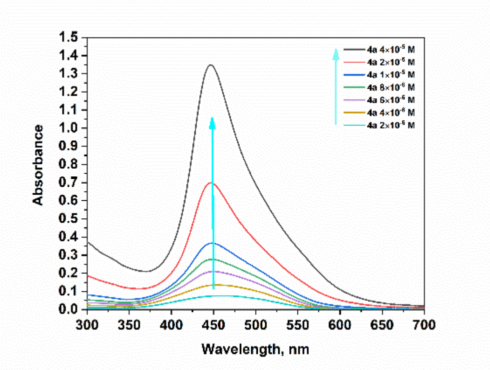

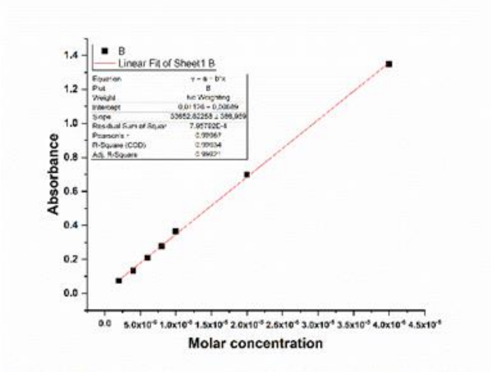

(**a**) **4a** (**b**) **4a**

Figure S1. UV/VIS spectra and calibration curve of dye **4a**, c= 0.2 – 4×10^{-5} M in TE buffer.

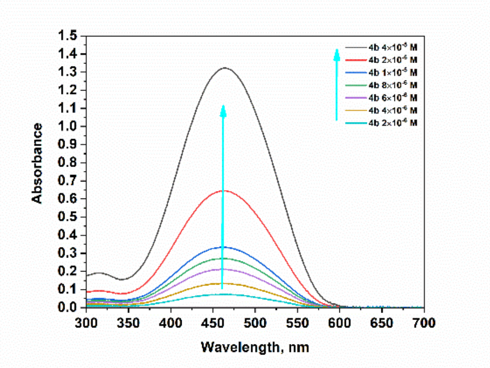

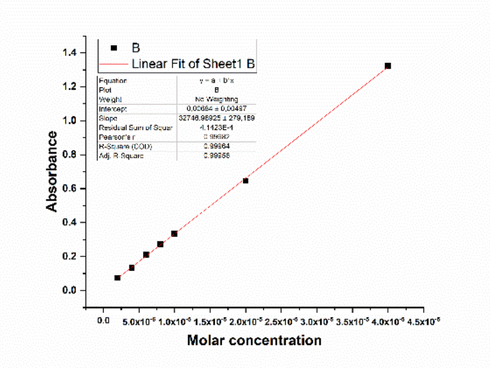

(**a**) **4b** (**b**) **4b**

Figure S2. UV/VIS spectra and calibration curve of dye **4b**, c= 0.2 – 4×10^{-5} M in TE buffer.

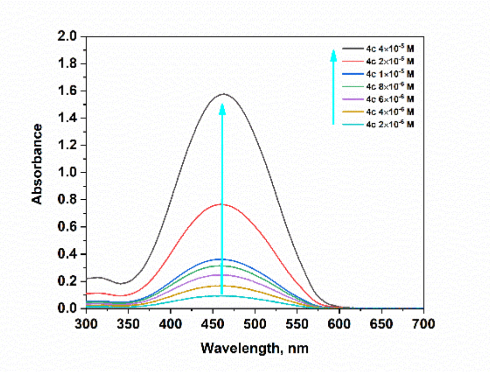

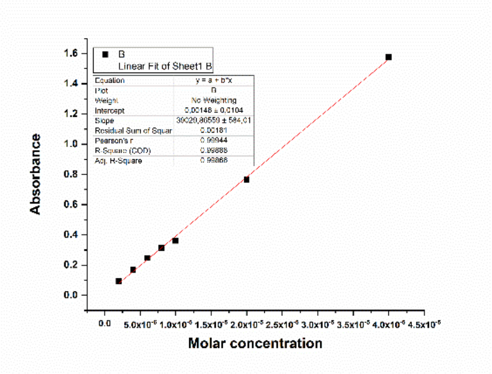

(**a**) (**b**)

Figure S3. UV/VIS spectra and calibration curve of dye **4c**, c= 0.2 – 4×10^{-5} M in TE buffer.

Geometry optimization of the trans isomers of compounds 4a-4c

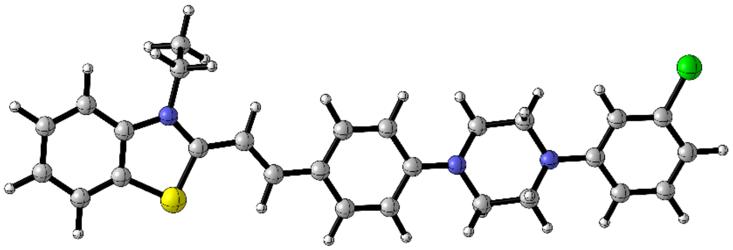

4a

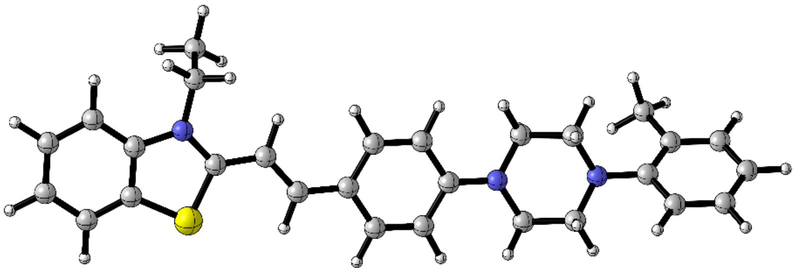

4b

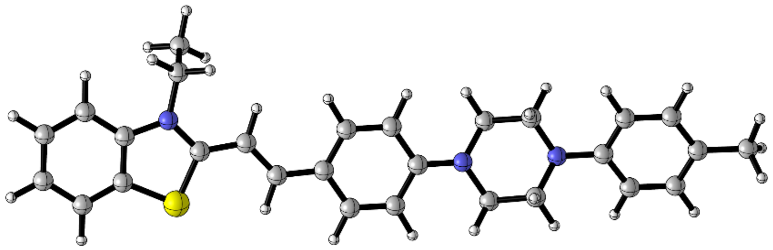

4c

Figure S4. B3LYP/6-31+G(d,p) optimized geometries of trans isomers of **4a**-**4c**. Color scheme: C–gray, S–yellow, H–white, N–blue.

Solvatochromic measurements in solvents with different polarity

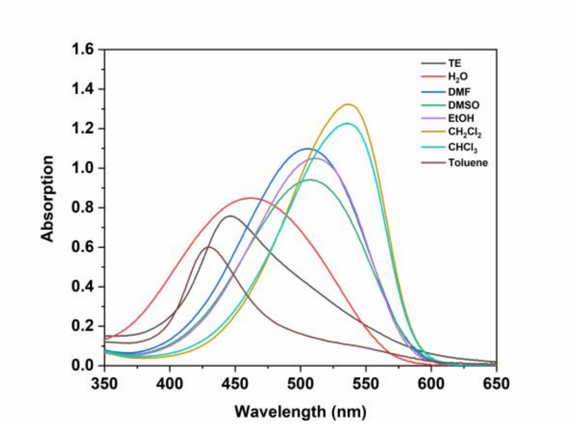

Figure S5. Position and intensity of the CT absorption maxima for dye **4a** (2×10^{-5} M) in solvents with different polarity.

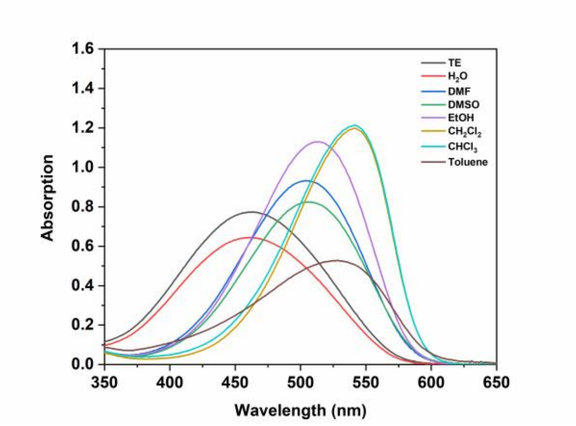

Figure S6. Position and intensity of the CT absorption maxima for dye **4b** (2×10^{-5} M) in solvents with different polarity.

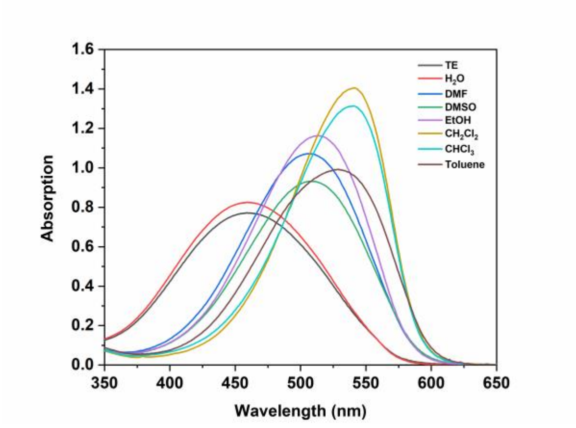

Figure S7. Position and intensity of the CT absorption maxima for dye **4c** (2×10^{-5} M) in solvents with different polarity.

Table S1. Kamlet–Taft parameters for different solvents (α, β, π*).

Solvent	α	β	π*
**Water**	1.17	0.47	1.09
**Ethanol**	0.86	0.75	0.54
**Dimethylformamide**	0.00	0.69	0.88
**Dimethyl sulfoxide**	0.00	0.76	1.00
**Dichloromethane**	0.13	0.10	0.82
**Chloroform**	0.44	0.00	0.58
**Toluene**	0.00	0.11	0.54

UV-vis spectra of dyes 4a and 4b in the presence of HCl

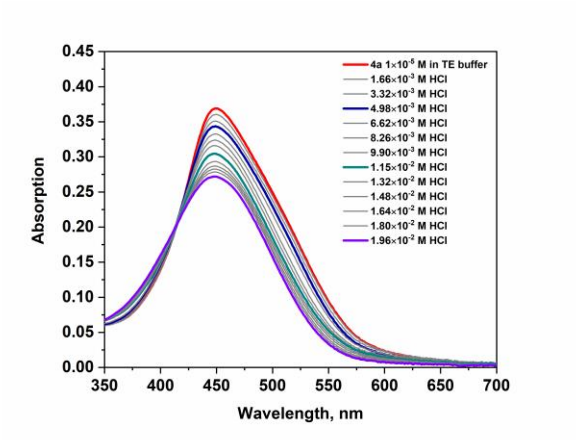

Figure S8. The changes in absorption spectrum of **4a** upon titration with HCl.

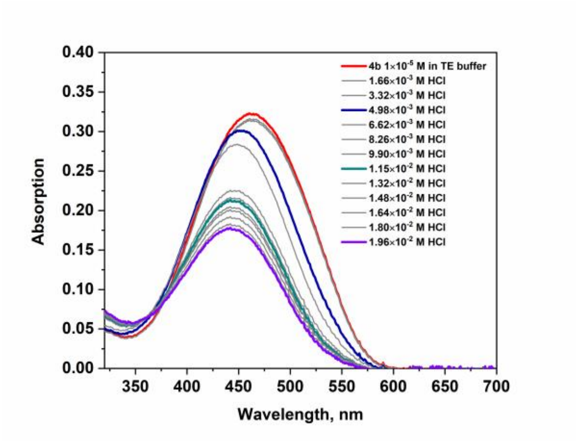

Figure S9. The changes in absorption spectrum of **4b** upon titration with HCl.

Absorption spectra of dyes 4a-4c in free state and in the presence of DNA and HCl

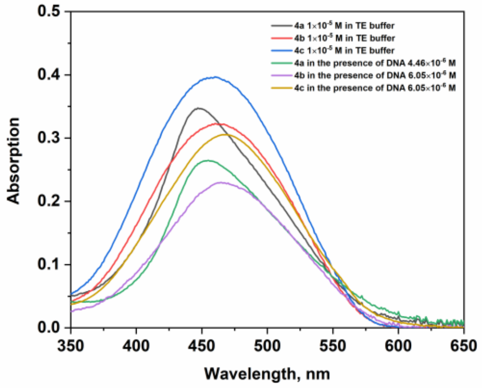

Figure S10. Absorption spectra of dyes **4a-4c** in solution and their complexes with DNA in TE buffer.

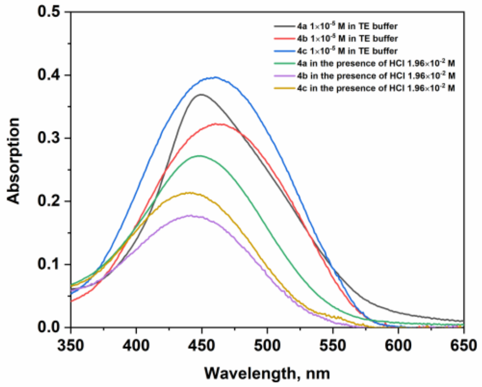

Figure S11. Absorption spectra of dyes **4a-4c** in solution and in the presence of HCl.

Fluorescence spectra of dyes 4a and 4b in solution and in the presence of DNA and HCl

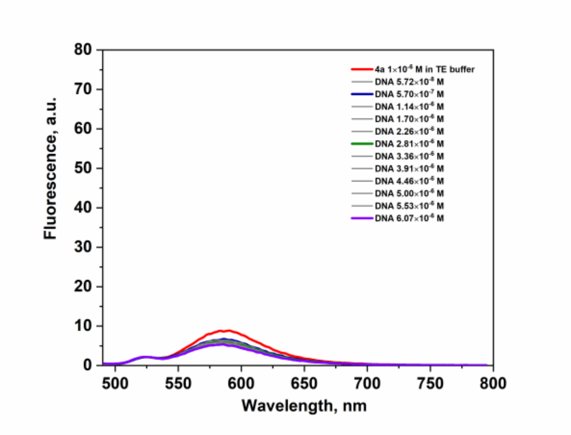

A)

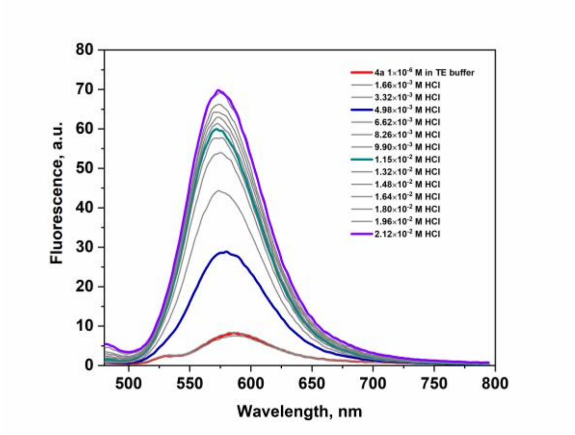

B)

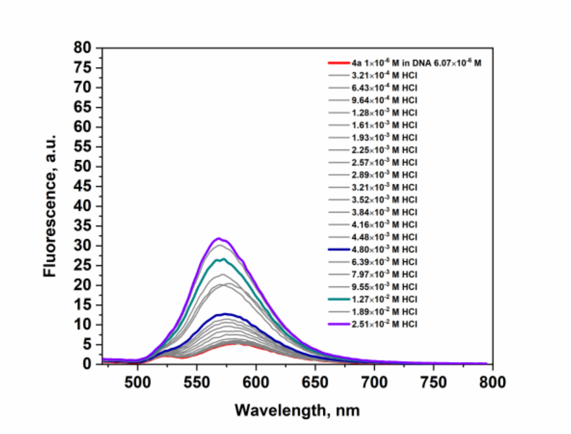

C)

Figure S12. Emission spectra of: **A) 4a** upon titration with DNA **B) 4a** upon titration with HCl; **C) 4a-dye-DNA complex** upon titration with HCl.

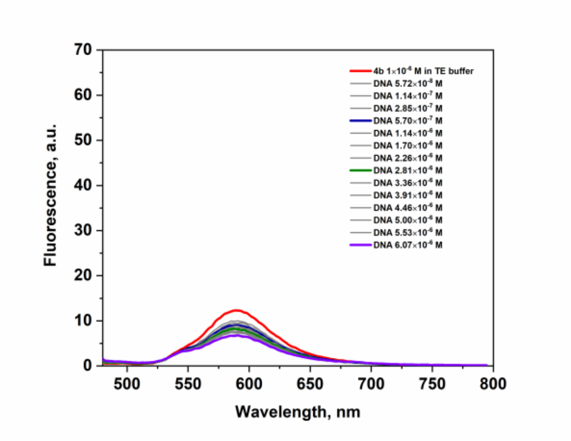

A)

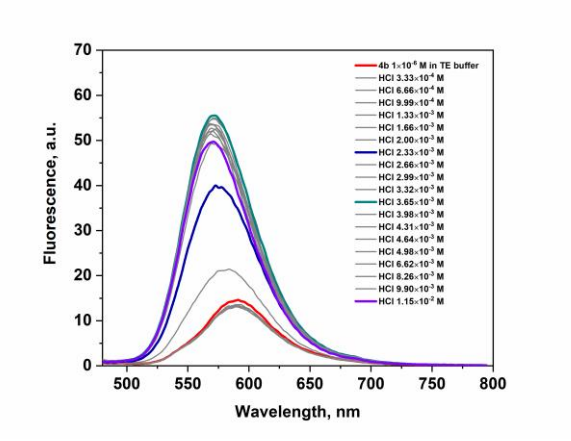

B)

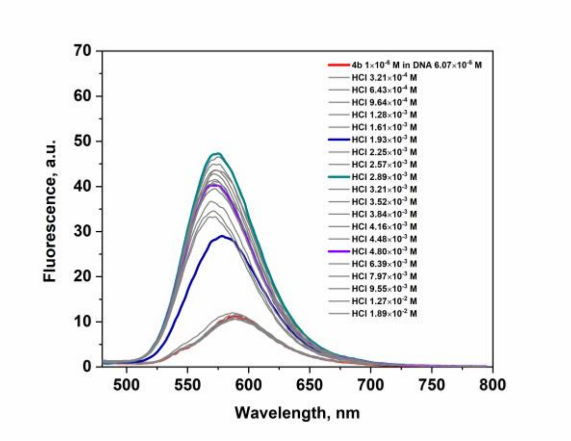

C)

Figure S13. Emission spectra of: **A) 4b** upon titration with DNA **B) 4b** upon titration with HCl; **C) 4b-dye-DNA complex** upon titration with HCl.

Molecular docking Table S2. Binding energy of the most favourable docking modes.

Conformer	Binding energy, kcal/mol, compound 4a	Binding energy, kcal/mol, compound 4b	Binding energy, kcal/mol, compound 4c	Binding energy, kcal/mol, thiazole orange
	**Groove binder**	**Groove binder**	**Groove binder**	**intercalator**
**1**	-9.4	-8.7	-9.2	-8.1
**2**	-8.6	-8.4	-8.5	-7.9
**3**	-8.4	-8.3	-8.4	-7.6
**4**	-8.4	-8.3	-8.4	-7.3
**5**	-8.1	-8.1	-8.2	-7.3
**6**	-8.1	-8.0	-8.0	-7.0
**7**	-8.1	-8.0	-8.0	-7.0
**8**	-8.0	-7.9	-7.9	-6.9
**9**	-7.9	-7.9	-7.8	-6.9

CD Spectra

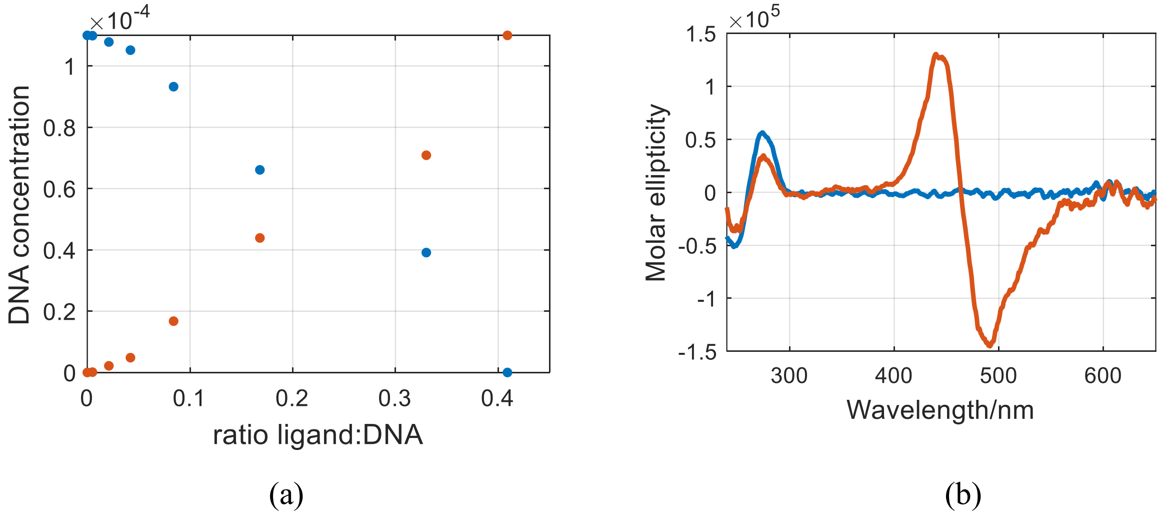

Figure S14. Deconvolution results of the CD spectra shown in Figure 11, obtained using Multivariate Curve Resolution based on Alternating Least Squares (MCR-ALS).

NMR characteristics

NMR spectra of dye **4a**

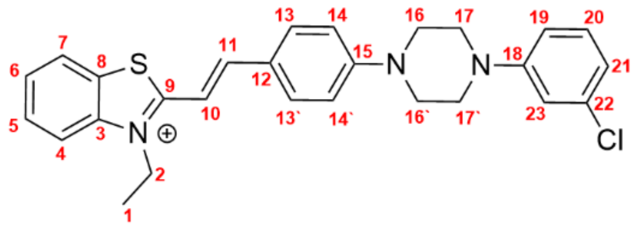

¹H-NMR:

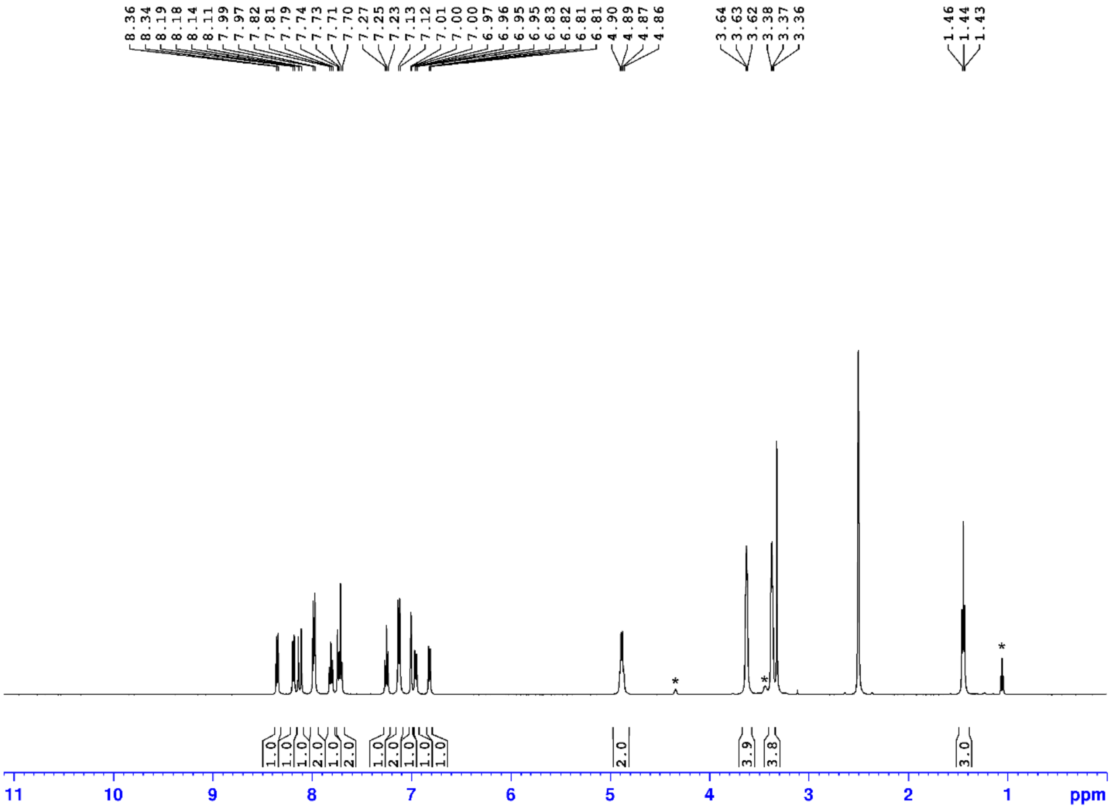

¹H-NMR spectrum of **4a**, residual solvent signals from ethanol are labelled with an asterisk (*)

¹H-NMR (DMSO-d_6, δ (ppm)): 1.44 (3H, t, H-1, *J*=7.1 Hz); 3.37 (4H, t, H-17, H-17`, *J*=5.0 Hz); 3.63 (4H, t, H-16, H-16`, *J*=5.1 Hz); 4.88 (2H, q, H-2, *J*=7.1 Hz); 6.82 (1H, dd, H-21, *J*=1.5 Hz, *J*=7.8 Hz); 6.96 (1H, dd, H-19, *J*=2.2 Hz, *J*=8.4 Hz); 7.00 (1H, t, H-23, *J*=2.0 Hz); 7.12 (2H, d, H-14, H-14`, *J*=9.0

Hz); 7.25 (1H, t, H-20, *J*=8.1 Hz); 7.70-7.74 (2H, m, H-5, *J*=7.7 Hz, H-11, *J*=15.3 Hz); 7.81 (1H, t, H-6, *J*=7.9 Hz); 7.98 (2H, d, H-13, H-13`, *J*=8.9 Hz); 8.12 (1H, d, H-10, *J*=15.3 Hz); 8.19 (1H, d, H-7, *J*=8.5 Hz); 8.35 (1H, d, H-4, *J*=8.0 Hz).

^{13}C-NMR:

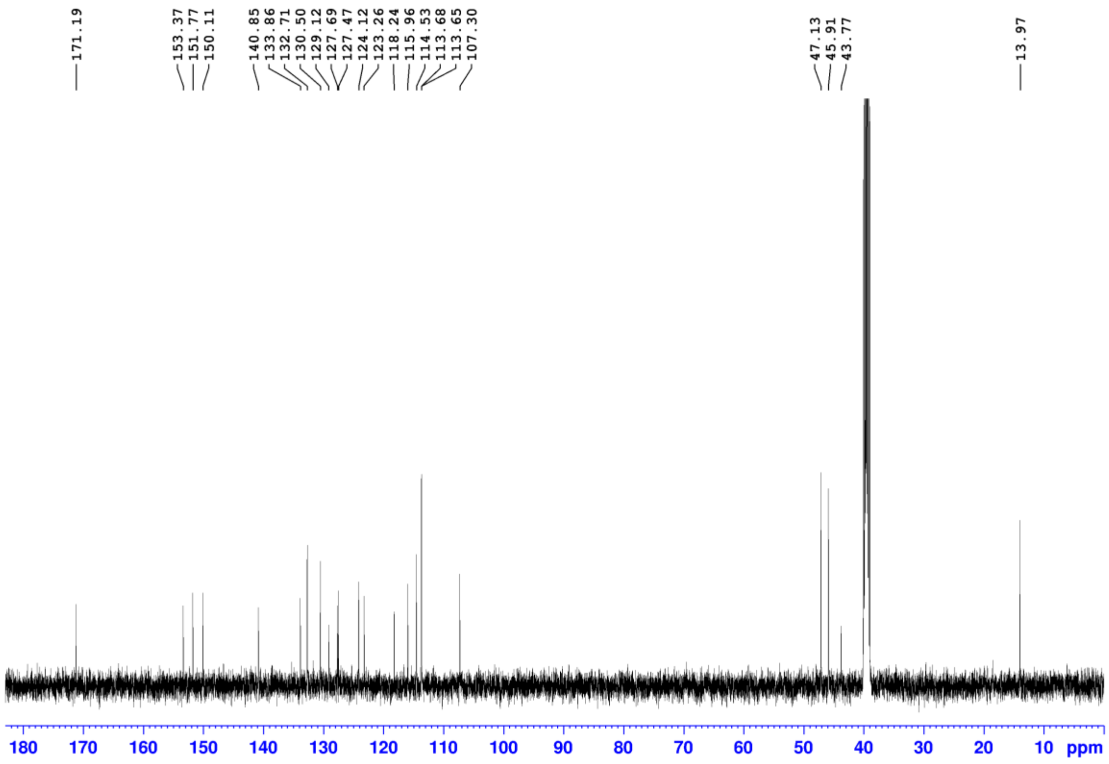

^{13}C-NMR (DMSO-d$_6$, δ (ppm)): 13.97 (1C, C-1); 43.78 (1C, C-2); 45.91 (2C, C-16, C-16`); 47.13 (2C, C-17, C-17`); 107.30 (1C, C-11); 113.65 (1C, C-19); 113.68 (2C, C-14, C-14`); 114.53 (1C; C-23); 115.96 (1C, C-7); 118.24 (1C, C-21); 123.26 (1C, C-12); 124.12 (1C, C-4); 127.47 (1C, C-5); 127.69 (1C, C-8); 129.12 (1C, C-6); 130.50 (1C, C-20); 132.71 (2C, C-13, C-13`); 133.86 (1C, C-18); 140.85 (1C, C-3); 150.11 (1C, C-10); 151.77 (1C, C-22); 153.37 (1C, C-15); 171.19 (1C, C-9).

HMBC:

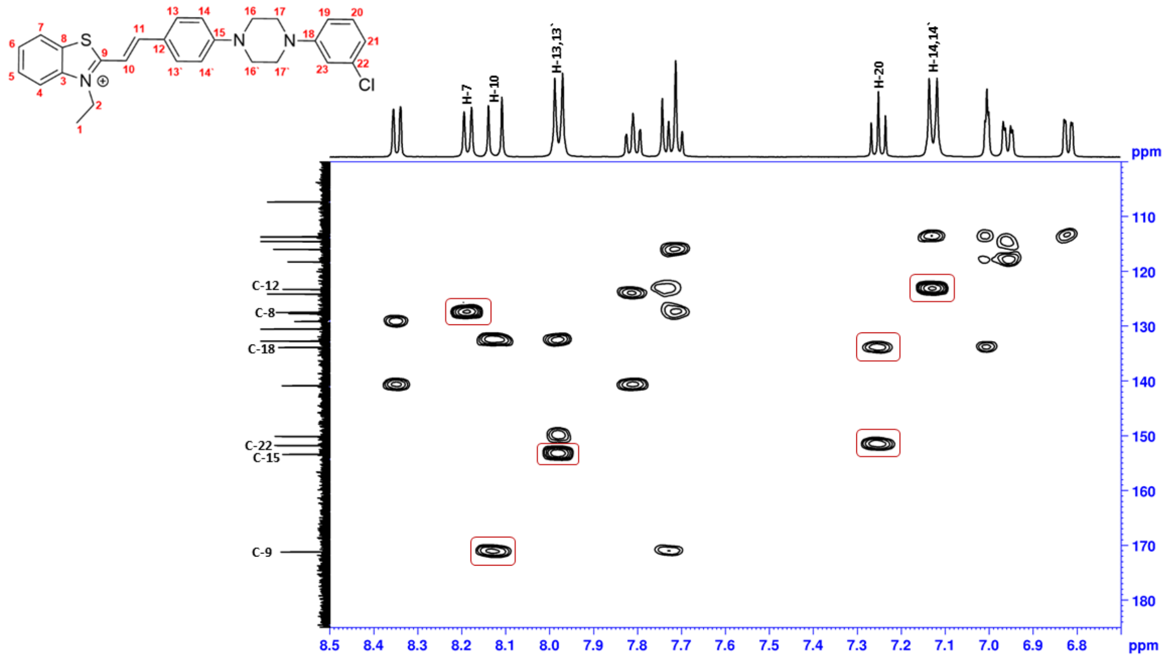

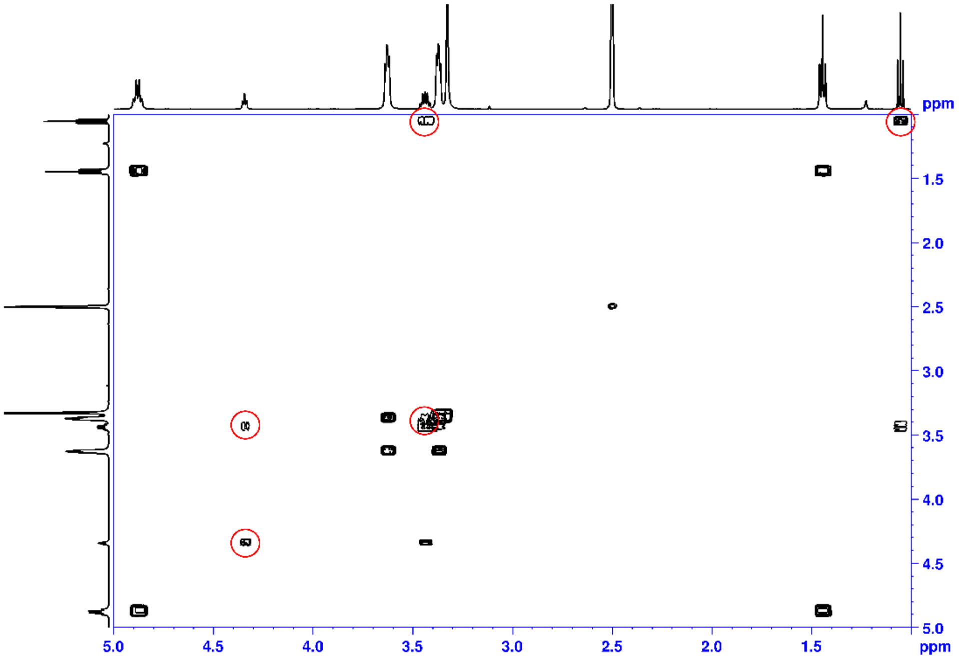

COSY spectrum of **4a**, residual solvent signals from ethanol are marked in red

NMR spectra of dye **4b**

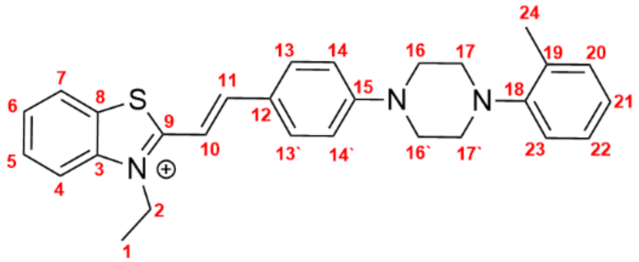

1H-NMR:

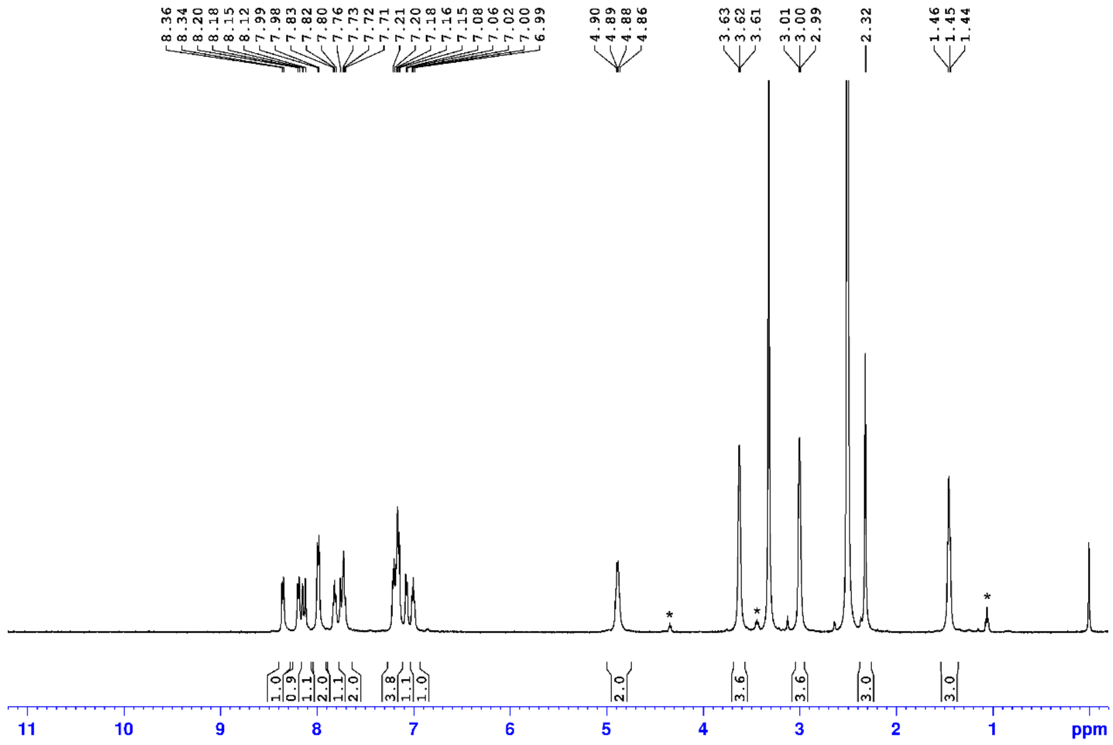

^{1}H-NMR spectrum of **4b**, residual signals from ethanol are are labelled with an asterisk (*)

^{1}H-NMR (DMSO-d6, δ (ppm)): 1.45 (3H, t, H-1, *J*=7.2 Hz); 2.32 (3H, s, H-24); 3.00 (4H, t, H-17, H-17`, *J*=4.5 Hz); 3.62 (4H, t, H-16, H-16`, *J*=4.4 Hz); 4.88 (2H, q, H-2, *J*=7.0 Hz); 7.00 (1H, t, H-21, *J*=7.4 Hz); 7.07 (1H, d, H-23, *J*=7.8 Hz); 7.14-7.21 (4H, m, H-14, H-14`, *J*=9.0 Hz, H-20, H-22); 7.70-7.75 (2H, m, H-11, *J*=15.6 Hz; H-5, *J*=7.6 Hz); 7.82 (1H, t, H-6, *J*=7.7 Hz); 7.98 (2H, d, H-13, H-13`, *J*=8.8 Hz); 8.13 (1H, d, H-10, *J*=15.4 Hz); 8.19 (1H, d, H-7, *J*=8.4 Hz); 8.35 (1H, d, H-4, *J*=8.0 Hz).

^{13}C-NMR:

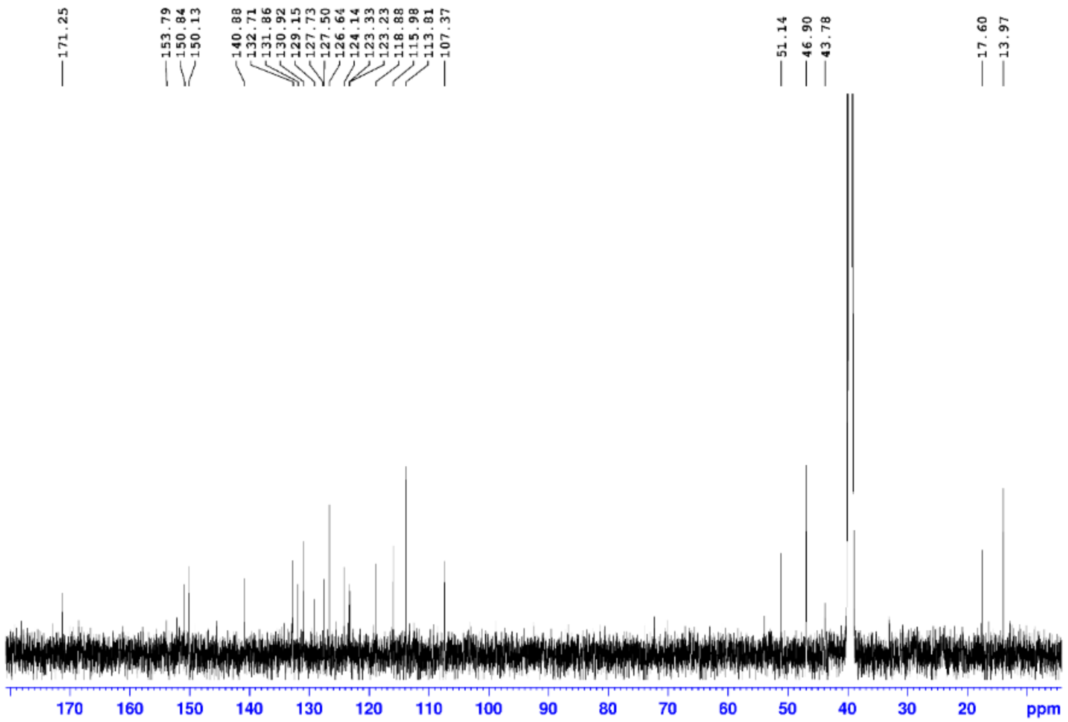

^{13}C-NMR (DMSO-d$_6$, δ (ppm)): 13.97 (1C, C-1); 17.60 (1C, C-24); 43.78 (1C, C-2); 46.90 (2C, C-16, C-16`); 51.14 (2C, C-17, C-17`); 107.37 (1C, C-11); 113,81 (2C, C-14, C-14`); 115.98 (1C, C-7); 118.88 (1C, C-23); 123.23 (1C, C-12); 123.33 (1C, C-21); 124.14 (1C, C-4); 126.64 (1C, C-20); 127.50 (1C, C-5); 127.73 (1C, C-8); 129.15 (1C, C-6); 130.92 (1C, C-22); 131.86 (1C, C-19); 132.71 (2C, C-13, C-13`); 140.88 (1C, C-3); 150.13 (1C, C-10); 150.84 (1C, C-18); 153.79 (1C, C-15); 171.25 (1C, C-9).

HMBC:

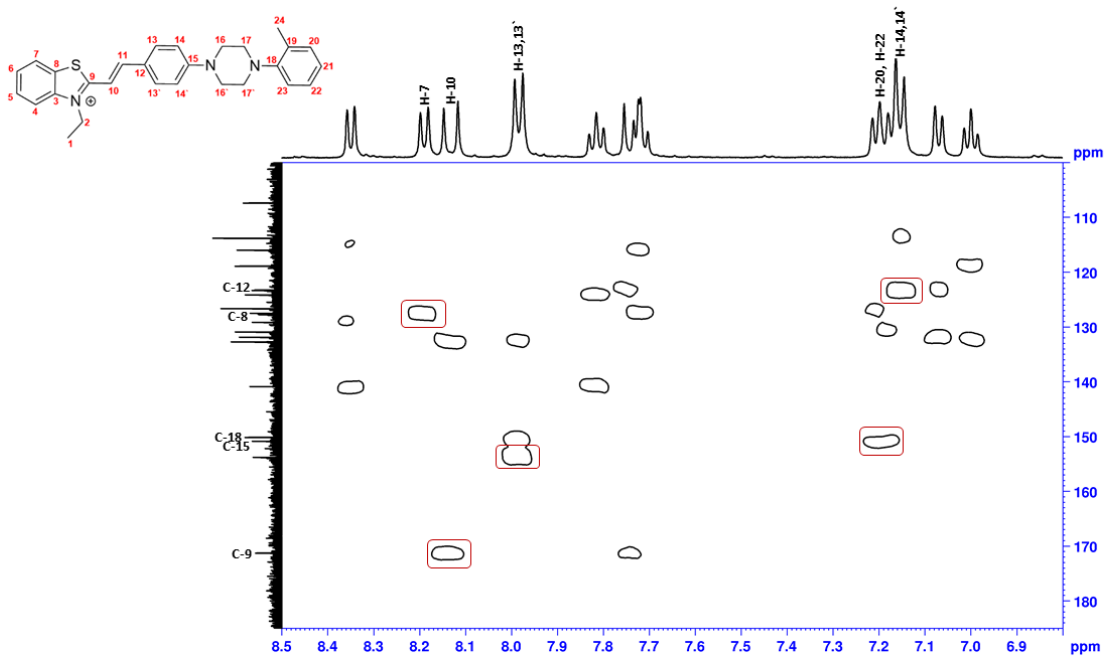

NMR spectra of dye **4c**

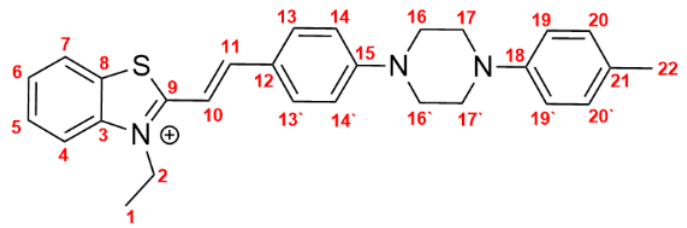

^{1}H-NMR:

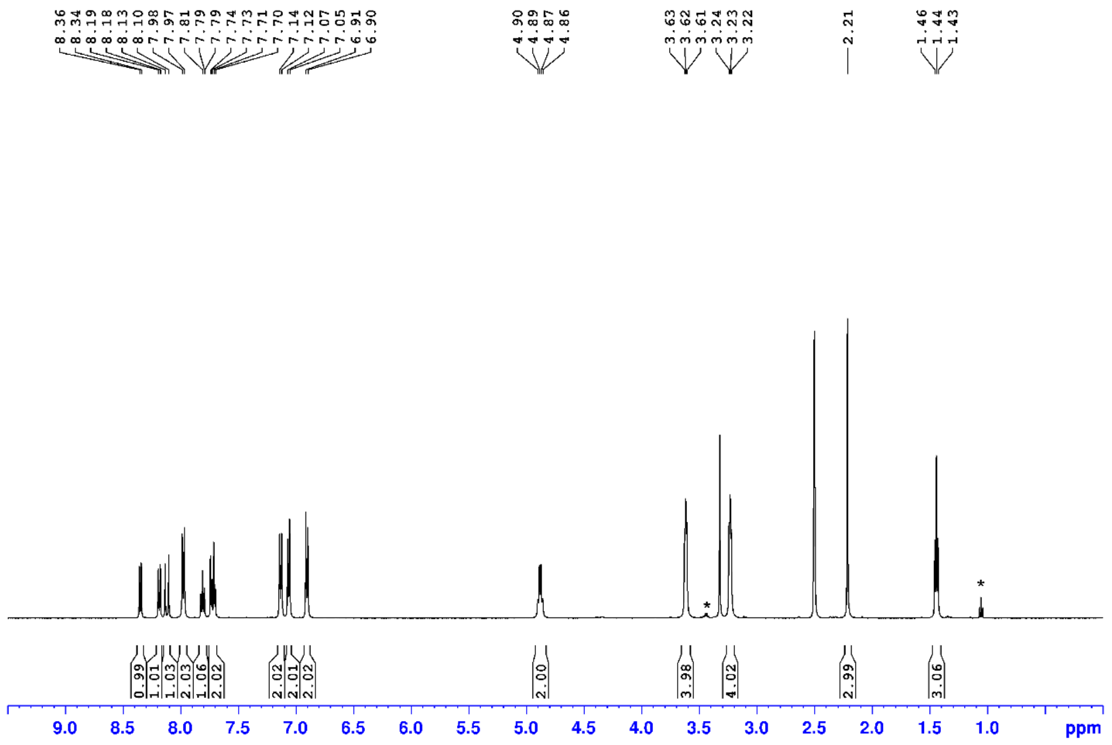

^{1}H-NMR spectrum of **4c**, residual signals from ethanol are labelled with an asterisk (*)

^{1}H-NMR (DMSO-d$_6$, δ (ppm)): 1.44 (3H, t, H-1, *J*=7.2 Hz); 2.21 (3H, s, H-22); 3.23 (4H, t, H-17, H-17`, *J*=5.1 Hz); 3.62 (4H, t, H-16, H-16`, *J*=5.1 Hz); 4.88 (2H, q, H-2, *J*=7.2 Hz); 6.90 (2H, d, H-19, H-19`, *J*=8.5 Hz); 7.06 (2H, d, H-20, H-20`, *J*=8.5 Hz); 7.13 (2H, d, H-14, H-14`, *J*=9.0 Hz); 7.70-7.74 (2H,

m, H-11, *J*=15.0 Hz, H-5, *J*=7.7 Hz); 7.8 (1H, dt, H-6, *J*=7.9 Hz, *J*=0.8 Hz); 7.98 (2H, d, H-13, H-13`, *J*=9.0 Hz); 8.12 (1H, d, H-10, *J*=15.3 Hz); 8.19 (1H, d, H-7, *J*=8.4 Hz); 8.35 (1H, d, H-4, *J*=8.0 Hz).

^{13}C-NMR:

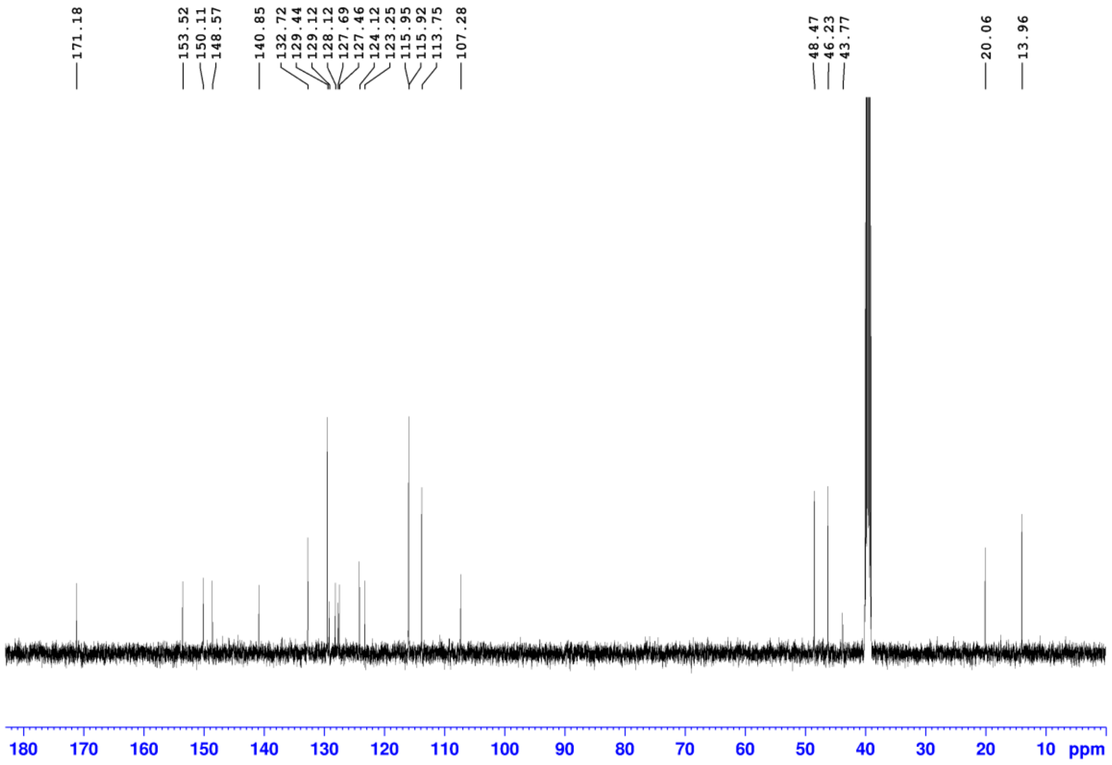

^{13}C-NMR (DMSO-d_6, δ (ppm)): 13.96 (1C, C-1); 20.06 (1C, C-22); 43.77 (1C, C-2); 46.23 (2C, C-16, C-16`); 48.47 (2C, C-17, C-17`); 107.28 (1C, C-11); 113.75 (2C, C-14, C-14`); 115.92 (2C, C-19, C-19`); 115.95 (1C, C-7); 123.25 (1C, C-12); 124.12 (1C, C-4); 127.46 (1C, C-5); 127.69 (1C, C-8); 128.12 (1C, C-21); 129.12 (1C, C-6); 129.44 (2C, C-20, C-20`); 132.72 (2C, C-13, C-13`); 140.85 (1C, C-3); 148.57 (1C, C-18); 150.11 (1C, C-10); 153.52 (1C, C-15); 171.18 (1C, C-9).

HMBC:

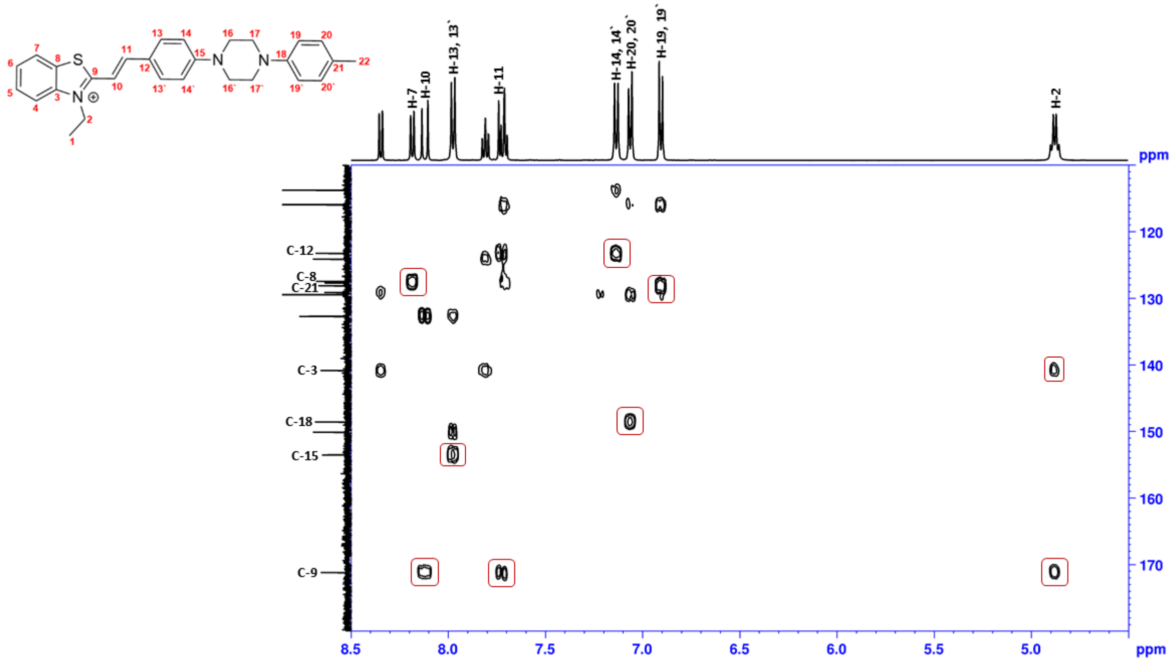

H-7
H-10
H-13, 13`
H-11
H-14, 14`
H-20, 20`
H-19, 19`
H-2
C-12
C-8
C-21
C-3
C-18
C-15
C-9
ppm
